# Structure-based screening for functional non-coding RNAs in fission yeast identifies a factor repressing untimely initiation of sexual differentiation

**DOI:** 10.1093/nar/gkac825

**Published:** 2022-10-19

**Authors:** Yu Ono, Kenta Katayama, Tomoki Onuma, Kento Kubo, Hayato Tsuyuzaki, Michiaki Hamada, Masamitsu Sato

**Affiliations:** Laboratory of Cytoskeletal Logistics, Department of Life Science and Medical Bioscience, School of Advanced Science and Engineering, Waseda University, 2-2 Wakamatsucho, Shinjuku-ku, Tokyo 162-8480, Japan; Laboratory of Cytoskeletal Logistics, Department of Life Science and Medical Bioscience, School of Advanced Science and Engineering, Waseda University, 2-2 Wakamatsucho, Shinjuku-ku, Tokyo 162-8480, Japan; Computational Bio Big-Data Open Innovation Laboratory (CBBD-OIL), National Institute of Advanced Industrial Science and Technology (AIST), 3-4-1 Okubo, Shinjuku-ku, Tokyo 169-8555, Japan; Laboratory of Cytoskeletal Logistics, Department of Life Science and Medical Bioscience, School of Advanced Science and Engineering, Waseda University, 2-2 Wakamatsucho, Shinjuku-ku, Tokyo 162-8480, Japan; Computational Bio Big-Data Open Innovation Laboratory (CBBD-OIL), National Institute of Advanced Industrial Science and Technology (AIST), 3-4-1 Okubo, Shinjuku-ku, Tokyo 169-8555, Japan; Bioinformatics Laboratory, Department of Electrical Engineering and Bioscience, School of Advanced Science and Engineering, Waseda University, 3-4-1 Okubo Shinjuku-ku, Tokyo 169-8555, Japan; Laboratory of Cytoskeletal Logistics, Department of Life Science and Medical Bioscience, School of Advanced Science and Engineering, Waseda University, 2-2 Wakamatsucho, Shinjuku-ku, Tokyo 162-8480, Japan; Computational Bio Big-Data Open Innovation Laboratory (CBBD-OIL), National Institute of Advanced Industrial Science and Technology (AIST), 3-4-1 Okubo, Shinjuku-ku, Tokyo 169-8555, Japan; Computational Bio Big-Data Open Innovation Laboratory (CBBD-OIL), National Institute of Advanced Industrial Science and Technology (AIST), 3-4-1 Okubo, Shinjuku-ku, Tokyo 169-8555, Japan; Bioinformatics Laboratory, Department of Electrical Engineering and Bioscience, School of Advanced Science and Engineering, Waseda University, 3-4-1 Okubo Shinjuku-ku, Tokyo 169-8555, Japan; Institute for Medical-oriented Structural Biology, Waseda University, 2-2 Wakamatsucho, Shinjuku-ku, Tokyo 162-8480, Japan; Laboratory of Cytoskeletal Logistics, Department of Life Science and Medical Bioscience, School of Advanced Science and Engineering, Waseda University, 2-2 Wakamatsucho, Shinjuku-ku, Tokyo 162-8480, Japan; Institute for Medical-oriented Structural Biology, Waseda University, 2-2 Wakamatsucho, Shinjuku-ku, Tokyo 162-8480, Japan; Institute for Advanced Research of Biosystem Dynamics, Waseda Research Institute for Science and Engineering, Graduate School of Advanced Science and Engineering, Waseda University, 3-4-1 Okubo, Shinjuku-ku, Tokyo 169-8555, Japan

## Abstract

Non-coding RNAs (ncRNAs) ubiquitously exist in normal and cancer cells. Despite their prevalent distribution, the functions of most long ncRNAs remain uncharacterized. The fission yeast *Schizosaccharomyces pombe* expresses >1800 ncRNAs annotated to date, but most unconventional ncRNAs (excluding tRNA, rRNA, snRNA and snoRNA) remain uncharacterized. To discover the functional ncRNAs, here we performed a combinatory screening of computational and biological tests. First, all *S. pombe* ncRNAs were screened *in silico* for those showing conservation in sequence as well as in secondary structure with ncRNAs in closely related species. Almost a half of the 151 selected conserved ncRNA genes were uncharacterized. Twelve ncRNA genes that did not overlap with protein-coding sequences were next chosen for biological screening that examines defects in growth or sexual differentiation, as well as sensitivities to drugs and stresses. Finally, we highlighted an ncRNA transcribed from *SPNCRNA.1669*, which inhibited untimely initiation of sexual differentiation. A domain that was predicted as conserved secondary structure by the computational operations was essential for the ncRNA to function. Thus, this study demonstrates that *in silico* selection focusing on conservation of the secondary structure over species is a powerful method to pinpoint novel functional ncRNAs.

## INTRODUCTION

Eukaryotic genomes are pervasively transcribed (1). Non-coding RNAs (ncRNAs) are transcripts which do not encode proteins. There are thousands of ncRNAs detected in the human genome (2–5). Although ncRNAs used to be recognized as junk molecules, recent studies reported some ncRNAs participating in cellular activities such as gene regulation (6). Nevertheless, there are few ncRNAs whose functions have been elucidated (7), and still a large portion of ncRNAs remain uncharacterized. Since those uncharacterized ncRNAs may play biological roles in cells, it is necessary to search for ncRNAs to identify those which are functional, to further elucidate the enigmatic landscape of ncRNA functions.

In general, ncRNAs are classified into two types by size: short ncRNAs (∼200 nt) and long ncRNAs (> 200 nt). Short ncRNAs include well-studied conventional ncRNAs such as tRNA, rRNA, snRNA, snoRNA and miRNA. The functions of long ncRNAs may be rather unconventional and diverse: for instance, XIST/Xist RNA, one of the earliest examples of functional ncRNAs, participates in X-chromosome inactivation (8). It covers one of the X-chromosomes in female cells and epigenetically induces gene silencing by recruiting chromatin-remodelling factors (9). In addition, several long ncRNAs are reported to participate in transcriptional or translational regulation by acting as a scaffold for protein complexes, or as an aptamer for specific proteins or mRNAs (10–12).

To identify functional ncRNAs further, a number of screenings were conducted. More than 16 000 human ncRNAs were systematically knocked-down in seven cell lines, to identify 499 that affect cell growth (13). However, knocking out each ncRNA for inspection is effective but laborious. Informatics-based screenings that rely on evolutionary conservation of ncRNAs over species have also been performed. Conservation of ncRNAs can be examined from the aspect of sequences, location and structures. First, nucleotide sequences of ncRNAs are conserved only weakly even in related species (14,15). Second, conservation of gene synteny on chromosomes would help to find functional ncRNAs. For example, physical co-localization of gene loci on the chromosome is occasionally conserved over species. Indeed, more ncRNAs are found conserved in location than in sequence (14,15). ncRNAs with a conserved location might participate in regulation of neighbouring genes (16). Third, ncRNAs form secondary structures via base pairing. Secondary structures could be considered as functional motifs of ncRNAs. In most cases, functional ncRNAs participate in biological processes through interaction with proteins, and this requires folding of the RNAs. For example, XIST RNA uses the AUCG tetraloop structure at the 5′ terminus to recruit polycomb repressive complex 2 (PRC2) (9).

Here, assuming that functional ncRNAs are structurally conserved, screenings that rely on the secondary structure may pick up potential functional ncRNAs. Computational programs that evaluate the structural conservation such as RNAz (17), EvoFold (18) and CMfinder (19) have been developed. Several studies have screened entire genomic DNA of bacteria, archaea, fungi and vertebrates for conserved RNA structures, although few of the predicted potential ncRNAs have been confirmed to play cellular roles (20–25).

The fission yeast *Schizosaccharomyces pombe* is widely used as a model to investigate cellular activities, taking advantage of easy genetic manipulation including gene knockout by use of polymerase chain reaction (PCR)-based gene targeting methods (26). *Schizosaccharomyces pombe* has a 12 Mb genome with >1800 annotated ncRNAs. There are some unconventional functional ncRNAs identified. For example, meiRNA, a meiosis-specific long RNA transcribed from the *sme2* gene locus, is known to be essential for sexual differentiation, particularly for progression of meiosis, as well as chromosome pairing (27–30). Some mRNA-type long ncRNAs controlling stress-regulated gene expression have also been identified (31,32). In addition, characterization of long ncRNAs include *rse1* (33), *SPNCRNA.1343* (16), *prt* (34) and *nam1* (35) has been reported (36).

Systematic deletion of long intergenic ncRNAs in fission yeast and their functional profiling have been recently conducted to present phenomics datasets for long ncRNAs (37). Screenings for functional ncRNAs have also been conducted previously based on sequence and location conservation, targeting annotated ncRNAs (15,16). For structure-based screening, an analysis targeting fungal genomes (>60 species) has predicted 15 structurally conserved ncRNA motifs, including one variant snoRNA motif which exists in *S. pombe* (22). However, no studies have conducted screening based on structural conservation for *S. pombe* ncRNAs, and only a few studies exist for the budding yeast *Saccharomyces cerevisiae* (20,25).

**Figure 1.**
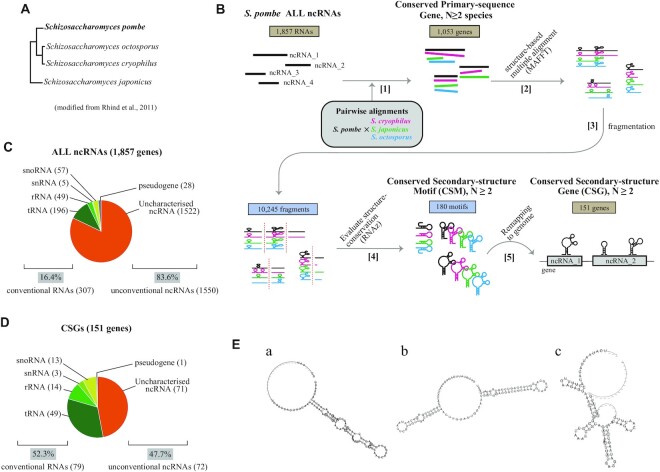
Structure-based analyses identify 151 ncRNAs conserved in secondary structure. (**A**) The phylogenetic tree of four *Schizosaccharomyces* species based on comparative genomics, adopted from a previous study (15). (**B**) The outline of the computational screening. Black, *S. pombe*; magenta, *S. cryophilus*; green, *S. japonicus*; blue, *S. octosporus*. [1] *S. pombe* 1857 ncRNAs were assessed for sequence similarity, and 1053 ncRNAs conserved in sequences with any of three *Schizosaccharomyces* species are selected as pairwise alignments. [2] Those were subjected to MAFFT assays to produce multiple alignments based on secondary structure conservation. [3] The alignments were digested into 10 245 fragments. [4] Conserved secondary structure motifs (CSMs, 180 motifs in total) were detected by RNAz. [5] CSMs were mapped onto the *S. pombe* genome to identify conserved secondary structure genes (CSGs; 151 genes in total). (**C**) Details of 1857 ncRNA genes in *S. pombe* used as input of the analyses. Numbers of genes in each category are shown in parentheses. Percentages of conventional ncRNAs (tRNA, rRNA, snRNA and snoRNA) and unconventional ncRNAs are shown below. (**D**) Details of 151 CSGs identified in (A). (**E**) Examples of CSMs in *SPNCRNA.1357* (a), *SPNCRNA.1145* (b) and *SPNCRNA.933* (c), detected in step [4] in (B). Predicted structures were drawn using the RNAalifold algorithm (72).

Here, we attempted to identify novel functional ncRNAs in *S. pombe* through combination of structure-based screening *in silico* and biological screening *in vivo*. In the first step, we designed *in silico* selection of structurally conserved ncRNAs among related species, based on comparative genomics among *S. pombe* and three other *Schizosaccharomyces* species: *S. cryophilus*, *S. japonicus* and *S. octosporus* (Figure 1A), as previously performed for budding yeast (25). Candidates were then subjected to the *in vivo* screening, in which we constructed *S. pombe* knockout mutants of the candidate ncRNAs and tested their phenotypic traits under several environmental and stress conditions. In this research, we screened a pool of 1857 annotated ncRNAs of *S. pombe* and nominated 151 ncRNAs through informatics. After their re-assessment, 14 of them were subjected to biological tests. Through the final assays, we found that an ncRNA named ‘nc1669’ is required for continuation of vegetative growth in *S. pombe* cells.

## MATERIALS AND METHODS

### Yeast strains and genetics

*Schizosaccharomyces pombe* strains used in this study are listed in Supplementary Table S1. The homothallic (*h*^90^) prototrophic strain JY3 was used as a prototrophic wild-type (WT) strain for all the experiments. KEN0040 and KEN0041 (*nc1669*Δ), as well as KEN0187 and KEN0188 (*nc1669FL*Δ), were constructed from JY3. MJ0006, MJ1551, SY50 and KEN0137 were used as controls in assays for drug sensitivity and cell viability.

Standard methods for *S. pombe* genetics were used (38). YE5S medium was used for growing cells. YE5S medium containing G418 (100 μg/ml) was used for selection of a strain having the *kan* gene that confers G418 resistance. SD–3S medium (SD medium containing yeast nitrogen base and glucose supplemented with adenine sulphate and leucine) was used for assays for the hyper-mating phenotype.

Gene deletion was performed using standard methods for PCR-based gene targeting (26). Oligonucleotide sequences for gene deletion and colony PCR are listed in Supplementary Table S2.

### Plate-based screening

For *in vivo* screening of deletion mutants of candidate genes, cells were grown overnight on YE5S agar plates, and then suspended in ddH_2_O to the concentration of 0.2 × 10^7^ cells/ml. Ten-fold serial dilutions of 10^7^–10^2^ cells were spotted on each assay plate. The assay plates are based on YE5S without or with thiabendazole (TBZ; 10–20 μg/ml; Sigma), hydroxyurea (HU; 5–10 mM; Sigma), H_2_O_2_ (1–5 mM; Wako), caffeine (7.5–12.5 mM; Sigma), CdSO_4_ (10–20 μg/ml, Sigma) or sorbitol (1–2 M; nacalai tesque). To test the UV sensitivity, 10-fold dilutions of cells were first spotted on YE5S agar plates, and then cells were irradiated with UV at 200 or 400 J/m^2^ with Stratalinker^®^ UV Crosslinker (Stratagene). The plates were then incubated at 26.5°C. Temperature sensitivity of deletion mutants was tested on YE5S agar plates incubated at 22, 26.5 and 36°C.

### Sexual differentiation assays

Sexual differentiation (mating, meiosis and sporulation) can be induced on SPA (sporulation agar), which does not contain nitrogen sources. Cells grown in YE5S were collected and spotted on SPA and incubated at 26.5°C for 10 or 24 h.

Mating in the presence of nitrogen (hyper-mating) was assayed on SD–3S agar plates: cells grown in YE5S were collected and suspended in a mixed solution of leucine and uracil to the concentration of 0.4 × 10^7^ cells/ml. The cell suspension (5 μl) was spotted onto SD–3S plates.

For observation of cells, cells were suspended to 1.6% formaldehyde (Thermo Fisher Scientific) and left to stand for 2 h. Samples were centrifuged to remove supernatants, and cells were washed with and resuspended in phosphate-buffered saline (PBS). Prior to observation, samples were subjected to mild sonication using an ultrasonic homogenizer VP-050N (TAITEC) for 45 s to dissolve cell aggregates. An aliquot (1 μl) of the cell suspension was mounted on a slide glass together with 1 μl of VECTASHIELD^®^ mounting medium with 4′,6-diamidino-2-phenylindole (DAPI; VECTOR laboratories) and covered with a coverslip.

Mating efficiency was calculated by the following formula: mating efficiency = 2 × number of zygotes/(2 × number of zygotes + number of unmated cells).

### Microscopy

Images of DIC (differential interference contrast) and fluorescence were acquired using the DeltaVision-SoftWoRx system (Applied Precision) (39). For fluorescence images, 12 sections with 0.4 μm intervals were taken along the *z*-axis, deconvolved and projected using the Quick Projection algorithm of the SoftWoRx software.

### RNA preparation and gene expression analyses

Cells grown in YE5S were dropped onto SD–3S plates as described above and incubated at 26.5°C for 48 h. Spots were collected and suspended in 1 ml of ice-cold diethylpyrocarbonate (DEPC)-treated water, spun down at 2000 rpm for 5 min at 4°C and supernatants were removed. Alternatively, for comparison of RNA levels depending on nitrogen availability in the medium (Figure 4), cells were cultivated in the presence (EMM + N) or absence (EMM–N) of the nitrogen source for 4 h and collected similarly.

The pellets were snap-frozen with liquid nitrogen and stored at –80°C overnight. The pellets were suspended in 520 μl of TES and 520 μl of phenol:chloroform:isoamyl alcohol 25:24:1 (pH 5.2) (nacalai tesque), incubated for 1 h at 65°C and placed on ice for 5 min. Suspensions were rotated at 14 000 rpm for 15 min at 4°C. Aqueous phases were transferred to new tubes with 400 μl of phenol:chloroform:isoamyl alcohol 25:24:1 (pH5.2). Aqueous phases were transferred to new tubes with 400 μl of chloroform and spun down at 14 000 rpm for 5 min at 4°C. Aqueous phases (∼300 μl) were transferred to new tubes, and 60 μl of 1.5 M sodium acetate (pH 5.3) and 750 μl of ice-cold 100% ethanol were added and stored at –20°C for 16 h. Samples were then rotated at 14 000 rpm for 20 min at 4°C, and supernatants were removed. After washing with 100 μl of 70% ethanol, samples were placed for 5 min at room temperature. Supernatants were then removed, and RNA pellets were dissolved in 50 μl of DEPC-treated water through repetitive freeze and thaw three times. Finally, the RNA samples were stored at –80°C.

DNAs contaminating RNA samples were eliminated by the TURBO DNA-free Kit (Invitrogen). A 5 μl aliquot of 10× Turbo DNase buffer, 1 μl of Turbo DNase, RNA extracts containing 8 μg of RNA and up to 50 μl of DEPC-treated water were mixed and incubated for 30 min at 37°C. DNase inactivation reagent (5 μl) was then added and incubated for 5 min at 25°C. Finally, the samples were centrifuged at 14 000 rpm for 5 min and ∼40 μl of supernatants were transferred to new tubes.

For reverse transcription, DEPC-treated water (1.2 μl), 10× Reverse transcription buffer (2 μl; Applied Biosystems), dNTPs (0.8 μl), 10× random primer or 1 μM strand-specific primer (Supplementary Table S2; 2 μl), reverse transcriptase (1 μl) and extracted RNA (2 μg) were mixed together and placed in the thermal cycler as follows: 25°C for 10 min, 37°C for 120 min and 85°C for 5 min, followed by cooling down at 4°C. For subsequent quantitative PCR, 5 μl of the samples were mixed with H_2_O (0.6 μl), THUNDERBIRD^®^ SYBR qPCR Mix (10 μl; Applied Biosystems), 3 μM forward primer (2 μl) (Supplementary Table S2), 3 μM reverse primer (2 μl) (Supplementary Table S2) and 50× ROX reference dye (0.4 μl) in a reaction plate. The RNA level was analysed with the StepOne Real Time PCR system (Applied Biosystems). Data were normalized with the expression level of the *act1* mRNA encoding *S. pombe* G-actin.

### Construction of plasmids

For expression of genes from plasmids, we modified the plasmid pREP1 as follows: the selection marker gene *LEU2* of pREP1 was replaced with the natMX6 marker cassette, to create pREP1-NatR. This was used because the host strain does not need to possess a leucine auxotroph in the genetic background that may affect mating ratios. For expression of full-length or truncated *nc1669* genes and *tRNAGlu08*, the *nmt1* promoter of pREP1-NatR was removed, and the genomic sequence of each ncRNA gene including the promoter and terminator regions was inserted, so that the inserted RNA genes can be natively expressed from their own promoters and terminators. For expression of the *nc1669* gene, the annotated gene region (2258 bp) flanked by the upstream 1153 bp and the downstream 1086 bp was cloned into pREP1-NatR without the *nmt1* promoter/terminator. The product is termed pREP1-NatR-nc1669FL (without P*nmt1*), from which the following truncated plasmids were derived: pREP1-NatR-nc1669woCSR and pREP1-NatR-nc1669FL-wo-nc1670. For expression of *tRNAGlu08*, the annotated gene region (72 bp) flanked by 1152 bp upstream and 919 bp downstream was inserted to replace P*nmt1*. This plasmid was termed pREP1-NatR-SPBTRNAGLU.08.

### Construction of a mutant with a shuffled sequence

The conserved secondary structure motif (CSM) sequence (117 nt) of *nc1669* was shuffled as follows. The sequence was randomly shuffled using uShuffle (40) to maintain the nucleotide composition. After randomization, an increase of the loop-forming probability was evaluated using ParasoR (41). The operations of uShuffle and ParasoR were repeated 50 650 times to compose the pipeline to nominate the most probable candidate sequence termed CSM-*shf1*. The loop formation probability of the original CSM sequence was 0.4496, and that of CSM-*shf1* was 0.5819, the highest possibility of loop formation of all the candidates. The sequence of CSM-*shf1* is shown in Supplementary Table S2.

The CSM-*shf1* DNA fragment was chemically synthesized, and was then introduced into JY3 (WT) cells to create the *nc1669* mutant harbouring the shuffled sequence using CRISPR/Cas9 [clustered regularly interspaced palindromic repeats (CRISPR)/CRISPR-associated peptide 9] methods. The pSR6 plasmid containing the crRNA sequence originated from the internal sequence of *nc1669* (Supplementary Table S2), and Cas9 (42) was constructed and introduced into JY3 cells together with the donor DNAs containing the CSM-*shf1* sequence. The genotype of transformants was confirmed by PCR. A pair of primers (Supplementary Table S2) was used to detect the CSM-*shf1* mutant sequence.

### Computational analyses

#### Extraction for ncRNAs and gene annotation

The *S. pombe* genome sequence and annotations were downloaded in the GenBank format from Pombase (https://www.pombase.org/data/genome_sequence_and_features/OLD/20170906/genbank/) (43,44) in June, 2016. All genes annotated as ‘misc_RNA’ were extracted with their gene names, their exonic DNA sequences and information on their genomic location. We defined ncRNAs as the sequences annotated in the ‘misc_RNA’ category, which is composed of conventional ncRNAs (tRNAs, rRNAs, snRNAs and snoRNAs), pseudogenes and unconventional ncRNAs that have not been characterized. The number of extracted ncRNAs was 1857 in total. Information on gene annotation was also downloaded from the Ensembl Fungi database, release 39 (https://fungi.ensembl.org/info/data/ftp/index.html) in the GFF3 format.

#### Pairwise alignments

Pairwise alignments of *S. pombe* sequences in comparison with each of three close *Schizosaccharomyces* species, namely ‘*Schizosaccharomyces cryophilus* versus *Schizosaccharomyces pombe*’, ‘*Schizosaccharomyces japonicus* versus *Schizosaccharomyces pombe*’ and ‘*Schizosaccharomyces octosporus* versus *Schizosaccharomyces pombe*’, were downloaded from the Ensembl Genomes database, release 86 (https://fungi.ensembl.org/info/data/ftp/index.html) (45), and the complete data were used without introducing any further cut-off by ourselves in the following procedures. All three alignments were constructed by the LastZ alignment algorithm (46). Using the pairwise alignments produced from the sequences of 1857 *S. pombe* non-coding genes as input, sequences that aligned to any of the related species were extracted. Genes that were not aligned to any sequences of the other three species, or genes aligned with sequences shorter than 50 bp were excluded in this process.

#### Structure-based screening

Structural analyses shown below were partly performed on the NIG supercomputer at ROIS National Institute of Genetics. RNAz software version 2.1 (17,47) was used to evaluate structural conservation of RNAs. RNAz requires alignment as input. It uses a support vector machine (SVM) learning algorithm to judge whether the input alignment is ‘structural RNA’ through comprehensive evaluation of the sequence similarity, structural similarity, structural stability and covariance base substitution. ‘Structural RNA’ has a defined secondary structure which is of functional importance and is conversed in the species used for comparison (47). For each input alignment, RNAz returns a *P*-value (range 0–1), which represents the probability of being a ‘structural RNA’.

The genes that were paired with genes in any of closely related species were defined as ‘Conserved Primary-sequence Gene’ in Figure 1B (1053 genes). Those were then applied to construction of structure-based multiple alignments with the MAFFT software (MAFFT v7.310) using the ‘Q-INS-i’ option (48). Then, alignments were digested into 120 nt fragments with a sequential 80 nt overlap with the rnazWindow.pl algorithm provided with the RNAz software. As represented in Figure 1B, 10 245 fragments were extracted by this procedure. Fragments of alignments were subjected to analysis with the RNAz software, using options ‘–forward, –no-shuffle, –cutoff = 0.5’. Note that only the forward direction of the alignments was used for evaluation. Alignments with a *P*-value >0.5 were extracted. Then, when applicable, neighbouring fragments were fused into one with the rnazCluster.pl algorithm, and fused regions were defined as CSMs (180 motifs were detected in total). Finally, *S. pombe* genes conserved within more than one of the species were defined as conserved secondary structure genes (CSGs).

#### Estimation of the false discovery rate

To estimate the number of false positives selected by RNAz, the following analyses were performed. After creating structure-based multiple alignments, the nucleotide sequence of an extracted *S. pombe* fragment in each alignment was randomly shuffled to destroy the secondary structure, using the random shuffling algorithm rnazRandomizeAln.pl provided by RNAz. Thereafter, shuffled alignments were subjected to similar RNAz analyses to evaluate their structure conservation. Consequently, 6.1% (64 out of 1053 input genes) of CSGs were found to be conserved even when their sequences were randomized. This indicates that those genes could be selected in a non-specific manner by RNAz, and the ratio (6.1%) has been defined as the false discovery rate (FDR).

## RESULTS

### Structure-based screening identified 151 ncRNAs evolutionarily conserved in closely related species

At the commencement of the *in silico* screening, 1857 ncRNAs have been annotated in the *S. pombe* genome in GenBank (June, 2016) (43). To identify functional ncRNAs, we selected ncRNAs which are structurally conserved in other *Schizosaccharomyces* species using computational programmes (Figure 1B).

The collection of 1857 non-coding gene sequences comprises tRNAs, rRNAs, snRNAs, snoRNAs, pseudogenes and uncharacterized ncRNAs (Figure 1C). The *in silico* part of the screen was carried out in the following steps (Figure 1B). See the Materials and Methods for details.

- Selection of ncRNAs with conserved primary sequences in closely related species ([1], Figure 1B). To identify conserved loci between *S. pombe* and at least another related species, pairwise alignments provided in the Ensembl Genome database (45) were referred to. This procedure selected 1053 *S. pombe* ncRNA genes.
- Structure-based alignment of the selected ncRNAs ([2], Figure 1B). The MAFFT software creates multiple alignments, considering secondary structures in addition to primary sequences of the input ncRNA sequences (48).
- Fragmentation ([3], Figure 1B). The multiple alignments obtained above were digested into 120 bases with a sequential 80 nt overlap, to produce 10 245 fragments.
- Selection of RNA motifs with conserved secondary structures in the species using the RNAz software (17,47) ([4], Figure 1B). As a result, 180 motifs (mean length = 147 nt) were extracted as conserved regions, which were then termed CSMs.
- Identification of conserved ncRNA genes in the species ([5], Figure 1B). The extracted CSMs were mapped onto the *S. pombe* genome, whose genes were defined as CSGs. The FDR, evaluating the accuracy of RNAz operations, was estimated as 6.1%, indicating that ∼64 out of 1053 genes could correspond to false positives.

Through the whole procedures, 151 genes were extracted as CSGs: ncRNAs with secondary structures that are conserved in *S. pombe* and at least another *Schizosaccharomyces* species (Figure 1D). These CSGs include 71 uncharacterized ncRNAs and 1 pseudogene (in total, 47.7% of CSGs), which are candidates for novel functional ncRNAs.

In the screen, we also included well-known functional ncRNAs (tRNAs, rRNAs, snRNAs and snoRNAs) to evaluate the efficiency and validity of the screening procedures. Our screening procedures highlighted 25.7% (79 out of 307) of those canonical ncRNAs (Figure 1C, D). Assuming that those well-known ncRNAs are generally conserved in sequence and structure over species, our computational screening could detect ∼25% of total functional ncRNAs. The ratio was defined as the DS (detection sensitivity). Well-known functional RNAs account for 16.4% of 1857 total non-coding genes in *S. pombe*, whereas they account for 52.3% of the total CSGs after the screen (Figure 1C, D). This finding indicates that our structure-based *in silico* screening has successfully pinpointed uncharacterized RNAs with conserved structures and sequences from the entire RNA pool.

Through the analyses, we identified many types of CSMs and found that those estimated structures varied among molecules. As can be seen in the examples shown in Figure 1E, some have simple linear structures (a and b), while some have complicated structures with multiple branching loops (c).

### Biological screening was performed to identify the functional ncRNA

**Figure 2.**
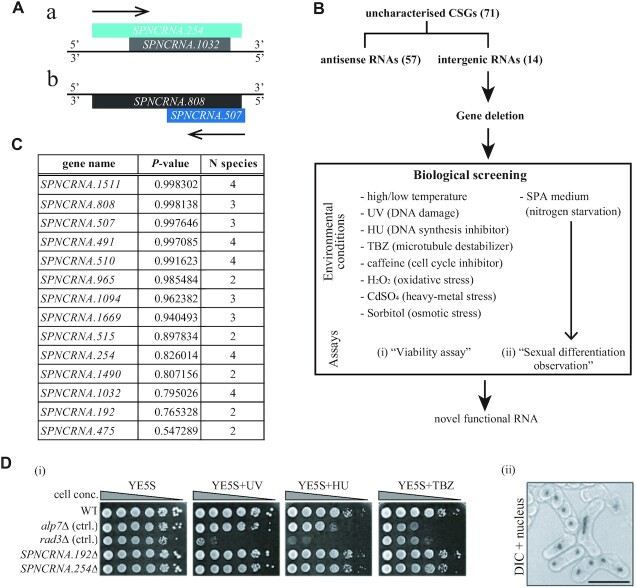
Plate-based knockout assays to identify functional ncRNAs. (**A**) Schematic diagrams of *SPNCRNA.254* and *SPNCRNA.1032* (a) and *SPNCRNA.507* and *SPNCRNA.808* (b). (**B**) The flow diagram illustrating the strategy from *in silico* to *in vivo* procedures. (**C**) List of structurally conserved RNAs that do not overlap with protein-coding sequences shown with *P*-values computed by RNAz and the number of species in which each RNA is structurally conserved. (**D**) Examples of biological screening. For viability assays (i), a 1/10 serial dilution of cells of the indicated knockout mutants was spotted on YE5S plates (26.5°C) without or with HU, TBZ or UV irradiation. The images are excerpts of the whole assays shown in Supplementary Figure S3. For observation of sexual differentiation (ii), cells were spotted on SPA, and ratios of mating, meiotic progression and spore formation were monitored. Nuclei were stained with DAPI and are shown merged with DIC. Scale bar, 10 μm.

To reveal the functions of 71 uncharacterized CSGs, those genes were listed for biological screening. Among them, 57 were antisense RNAs that overlap with protein-coding genes on their complementary strand, and 14 were ncRNAs that did not overlap with protein-coding sequences on either strand (Figure 2A–C). We focused on the ncRNAs without protein CDS overlaps. This is mainly for a practical reason, i.e. to achieve an efficient high-throughput *in vivo* screen. Knockout of ‘antisense RNA’ genes inevitably removes protein-coding genes located on the complementary strand simultaneously. This obscures which gene is responsible for the observed phenotype. In addition, antisense RNAs might often be predicted to regulate expression of the genes located on the sense strand.

Many of 14 candidates overlapped with other ncRNA(s) in either strand, and some overlapped with untranslated regions (UTRs) of protein-coding genes. Candidate genes *SPNCRNA.254* and *SPNCRNA.507* overlap with other candidates *SPNCRNA.1032* and *SPNCRNA.808*, respectively, and only one mutant lacking overlapping two genes was constructed for each case (Figure 2A). In total, 12 knockout mutants were constructed and subjected to the biological screening.

In the biological screen, mutant cells were placed under a number of environmental conditions to examine phenotypes during mitotic and meiotic cycles and in reaction to external stresses (Figure 2B). These include growth assays at high or low temperature, a reaction to UV irradiation that induces DNA damage, in the presence of HU as an inhibitor of DNA synthesis, thiabendazole (TBZ) as an anti-mitotic drug destroying microtubules, caffeine that affects cell cycle progression, H_2_O_2_ as an oxidative stress, CdSO_4_ for a heavy metal stress and sorbitol for osmotic stress (Figure 2B, D; Supplementary Figures S1–S7). In addition, mutant cells were observed under nitrogen starvation conditions. *Schizosaccharomyces pombe* undergoes sexual differentiation (mating followed by meiosis and sporulation) upon nitrogen starvation. To monitor phenotypes there, cells were cultured in media lacking nitrogen sources and the cell morphology was observed under a microscope (Figure 2D). Mating efficiencies of the knockout mutants were quantified (Supplementary Figure S8).

In the plate-based assays, there were some knockout mutants that showed phenotype, albeit partially. The deletion mutant of *SPNCRNA.491*Δ was moderately sensitive to 10 μg/ml TBZ (Supplementary Figures S2–S4), implying that ncRNA491 may be related to microtubule functions or regulation.

### nc1669 regulates untimely initiation of sexual differentiation

**Figure 3.**
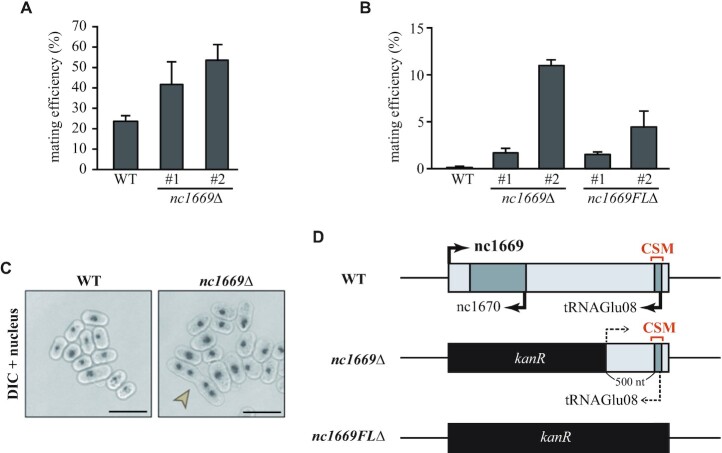
nc1669 is required for timing of sexual differentiation. (**A**) Mating efficiencies of WT and *nc1669*Δ cells under nitrogen starvation (on SPA plates, 10 h). Two independent colonies, denoted as #1 and #2, of the identical genotype were tested. (**B**, **C**) Mating efficiencies in the nitrogen-rich synthetic medium (SD–3S) were counted for WT, *nc1669*Δ and *nc1669FL*Δ cells (B). In (B), two independent colonies were chosen. Merged images of DIC and DAPI (nuclei) for WT and *nc1669Δ* cells (C). Scale bars, 10 μm. (**D**) Schematic diagrams of the *nc1669* gene locus in WT, *nc1669*Δ and *nc1669FL*Δ strains. The black boxed regions of the gene were replaced with the marker gene conferring G418 resistance (*kanR*). The position of the CSM identified in the screen is shown. Arrows, direction of transcription. Dotted arrow, not clear if transcription occurs therefrom.

The plate-based screening highlighted the knockout mutant of *SPNCRNA.1669* (*nc1669*Δ), which showed higher mating efficiency than WT cells under nitrogen starvation (Figure 3A). Assuming that *nc1669*Δ cells are competent to easily undergo sexual differentiation, we hypothesized that the mutant cells may conjugate even in the presence of nitrogen sources. When cells were grown in media containing nitrogen sources, some of the *nc1669*Δ cells underwent mating (followed by meiosis), whereas WT cells never initiated mating (Figure 3B, C). Multiple clones, denoted as #1 and #2, of the same genotype were subjected to phenotype analyses. The penetrance of the phenotype was different between clones (Figure 3A, B). This tendency remained after genetic backcrossing, excluding the possibility that additional mutations unexpectedly occurred in some clones.

Untimely sexual differentiation in the presence of nitrogen sources is commonly called ‘hyper-mating’. The phenotype was seen in the *nc1669*Δ mutant originally created for the initial screen. It is of note that the *nc1669*Δ mutant still harbours the CSM sequence in the locus, which has been identified as a conserved motif over species by the bioinformatic screen as shown above (Figure 3D). The CSM overlaps with *tRNAGlu08* in the antisense strand.

**Figure 4.**
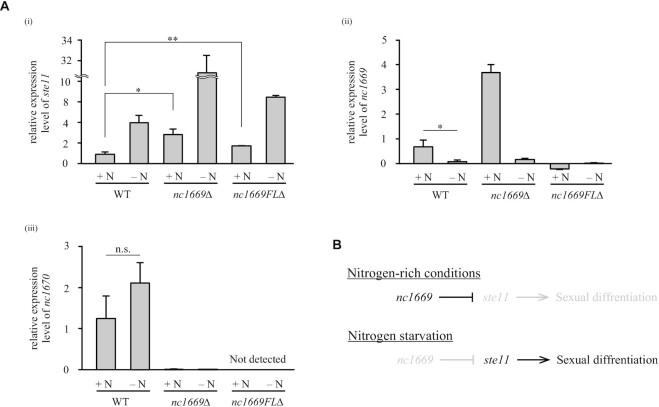
nc1669 represses expression of *ste11* in the presence of nitrogen. (**A**) Expression levels of *ste11* (i), *nc1669* (ii) and *nc1670* (iii) in the indicated strains, cultivated in the presence (+N) or absence (–N, 4 h) of the nitrogen source, were quantified and normalized with the *act1* mRNA level. Average ± SEM (three trials). *Pp = 0.0149, **P = 0.00934 (i), *P = 0.0282 (ii), n.s., not significant, *P* = 0.0685 (iii) (Student's *t*-test). (**B**) Cascade of sexual differentiation in nitrogen-rich conditions and under nitrogen starvation.

To examine the relationship between *nc1669* and sexual differentiation, the expression level of *nc1669* was monitored in response to nitrogen starvation (Figure 4A). For reference, expression of *ste11* was examined, which encodes a master transcription factor for sexual differentiation and is transcriptionally up-regulated in response to nitrogen starvation (49) (i, Figure 4A). An almost comparable high level of *ste11* was detected, even in the presence of nitrogen, in the *nc1669Δ* mutant (i, Figure 4A). Up-regulation of *ste11* expression in *nc1669*Δ indicates that the ncRNA nc1669 negatively regulates expression of *ste11* in vegetative WT cells (Figure 4B). In line with this, the expression of *nc1669* was reduced in response to nitrogen starvation (ii, Figure 4A).

**Figure 5.**
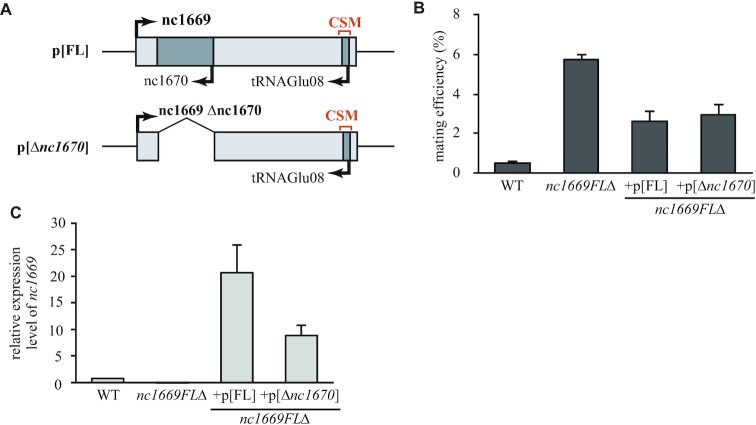
nc1670 is not responsible for the hyper-mating phenotype. (**A**) Schematic diagrams of the insert regions expressed in plasmids. (**B**) Mating efficiency in the nitrogen-rich medium SD–3S + Nat. Average ± SEM (three trials). *n* >1400 cells. (**C**) Expression levels of *nc1669* in the indicated strains were quantified and normalized with the *act1* mRNA level. Average ± SEM (three trials).

Although the *nc1669*Δ mutant lacks most of the gene, the remaining portion appeared to be highly expressed, possibly due to artificial effects due to insertion of the marker gene *kanR* (ii, Figure 4A). The remaining transcripts appeared non-functional (loss of function), because the null (complete deletion) mutant *nc1669FL*Δ (Figure 3D) exhibited the hyper-mating phenotype similarly to the *nc1669*Δ mutant (Figure 3B). Both *nc1669*Δ and *nc1669FL*Δ mutants lack another ncRNA, *SPNCRNA.1670* (*nc1670*), that overlaps with *nc1669* in its antisense strand (Figure 3D). First, the expression level of *nc1670* did not significantly change in response to nitrogen starvation (iii, Figure 4A). In order to determine deletion of which ncRNA is responsible for the hyper-mating phenotype, two plasmids were constructed: the plasmid p[FL] contains and expresses the full length of the *nc1669* gene, whilst the other one p[Δ*nc1670*] expresses the partial *nc1669* lacking the *nc1670* region (Figure 5A). Those plasmids were introduced into the *nc1669FL*Δ strain, and we found that both transformants complemented the hyper-mating phenotype to a similar degree (Figure 5B, C). These results demonstrated that the lack of *nc1669*, but not of *nc1670*, was the cause of the hyper-mating phenotype.

In summary, we identified the ncRNA nc1669 as an uncharacterized functional ncRNA that possesses a CSM, which is required to negatively regulate initiation of untimely sexual differentiation.

### Investigation of molecular function of nc1669

**Figure 6.**
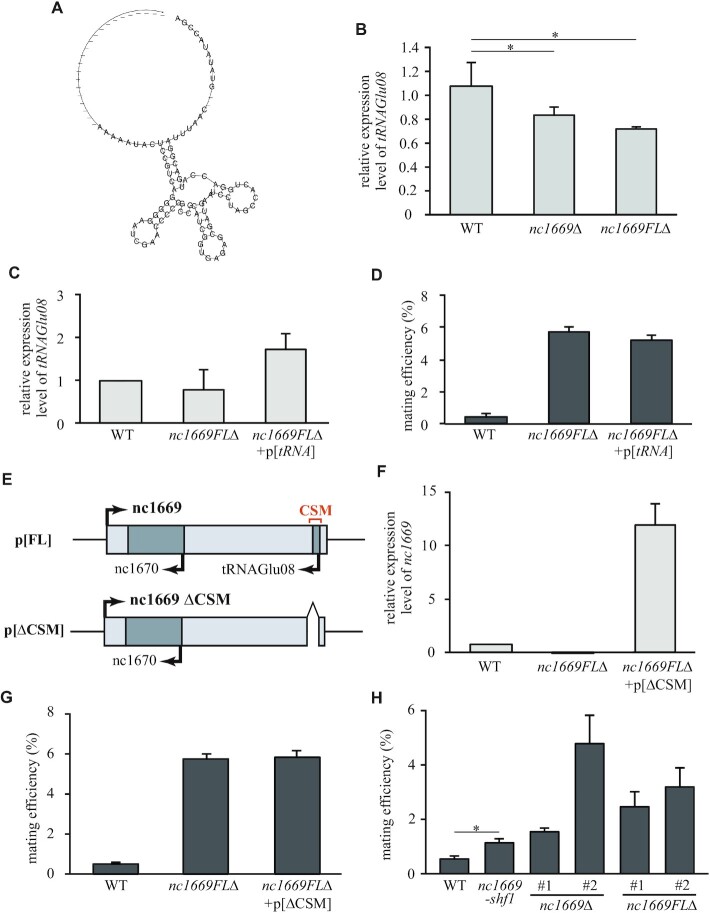
The CSM, but not its antisense tRNA, is required for the function of nc1669. (**A**) Secondary structure of the CSM in *nc1669* predicted by RNAalifold. (**B**) Expression levels of *tRNAGlu08* in the indicated strains were quantified and normalized with the *act1* mRNA level. Average ± SEM (three trials). *p = 0.0491 (between the WT and *nc1669*Δ), 0.0378 (between the WT and *nc1669FL*Δ) (Student's *t*-test). (**C**) Relative expression levels of *tRNAGlu08* in the indicated strains. Average ± SEM (three trials). (**D**) Mating efficiencies of WT, *nc1669FL*Δ and *nc1669FL*Δ+p[*tRNA*] cells in nitrogen-rich medium (SD–3S + Nat). Average ± SEM (three trials). (**E**) Regions cloned in expression plasmids are shown. The plasmid p[FL] contains the full length of *nc1669*, whereas p[ΔCSM] contains *nc1669* lacking the CSM. (**F**) Relative expression levels of *nc1669* in the indicated strains. Average ± SEM (three trials). (**G**) Mating efficiencies of the indicated strains on nitrogen-rich medium (SD–3S + Nat). Average ± SEM (three trials). *n* >1000 cells. (**H**) Mating efficiencies of WT, *nc1669-shf1*, *nc1669*Δ and *nc1669FL*Δ cells in nitrogen-rich medium (SD–3S). Average ± SEM (four trials). *n* >2500 cells. *p = 0.0252 (χ^2^ test).

Those experiments demonstrate that the ncRNA nc1669 represses untimely mating in nutrition-rich conditions. We further examined how the ncRNA nc1669 represses hyper-mating. From the antisense strand of *nc1669*, tRNA encoding glutamic acid (*tRNAGlu08*, *SPBTRNAGLU.08*) is transcribed (Figure 3D). In the chromosome, the CSM of *nc1669* overlaps with the *tRNA* gene on the antisense strand, and the CSM also displayed a tRNA-like structure comprising three hairpin loops (Figure 6A).

This raises the question of whether the CSM of nc1669 may just reflect an inversion of the structure of overlapping tRNAGlu08. However, our screen did not highlight tRNAGlu08 as a conserved ncRNA. Even if an RNA forms a secondary structure, it does not necessarily mean that the antisense RNA also forms a secondary structure. This is partly due to U–G forming a base pair that may constitute a secondary structure, whereas its antisense bases C–A do not form a pair in RNAs. Indeed, the RNAz operation evaluated that the degree of structure conservation was extremely low in tRNAGlu08 (the RNA class probability *P* = 0.07), whereas it was quite high in the nc1669 coding strand (*P* = 0.94). This indicates that the CSM of nc1669 is not just a reflected structure of tRNAGlu08.

Several studies suggest that tRNAs and related factors regulate sexual differentiation in yeast (50,51). We therefore postulated that nc1669 may cooperate with tRNAGlu08 to repress untimely mating of cells. To test this possibility, we first monitored the amount of tRNAGlu08 in *nc1669*Δ and in *nc1669FL*Δ cells. In the partial deletion of *nc1669* that still retains the sequence for the *tRNAGlu08* gene, the expression level of *tRNAGlu08* was reduced to a similar extent to that in *nc1669FL*Δ cells lacking the tRNA gene (Figure 6B). This indicates that the partial deletion of the *nc1669* gene affected expression of the neighbouring *tRNAGlu08* gene. It is possible that unexpected up-regulation of partial nc1669 RNAs (ii, Figure 4A) affected silencing of the antisense *tRNAGlu08*.

To further investigate whether the untimely mating phenotype of *nc1669*Δ is due to the loss of *tRNAGlu08* expression, we prepared the *nc1669FL*Δ strain harbouring the plasmid p[*tRNA*] that contains the *tRNAGlu08* gene. Although expression of *tRNAGlu08* was complemented by plasmids in the *nc1669FL*Δ mutant (*nc1669FL*Δ+p[*tRNA*], Figure 6C), untimely mating was still observed (Figure 6D). Through these results, we conclude that the hyper-mating phenotype was due to the loss of *nc1669per se*, and not through regulation of *tRNAGlu08*. Having clarified that tRNAGlu08 is unrelated to the hyper-mating phenotype, we next investigated whether the CSM in nc1669 is essential for the function. When *nc1669* without *nc1670* was expressed in the *nc1669FL*Δ strain, untimely mating was complemented in the same way as with the full-length *nc1669* (Figure 5). This indicates that the remaining region containing the CSM in *nc1669* retains the ability to repress untimely mating. Finally, when we induced expression of *nc1669* without the CSM in the *nc1669FL*Δ strain (*nc1669FL*Δ+p[ΔCSM], Figure 6E), we found that the exogenous construct did not retain the ability to repress the hyper-mating phenotype (Figure 6F, G).

To further strengthen this result, we also prepared the *nc1669-shf1* mutant, in which the nucleotide sequence of the CSM region was randomly shuffled so that the original secondary structure of *nc1669* was substantially altered by maximizing the possibility of loop formation among all the candidates (Supplementary Figure S9A). The *nc1669-shf1* mutant showed the phenotype of untimely mating (Figure 6H), which may be ascribed to a moderate increase in *ste11* expression (i, Supplementary Figure S9B). The phenotype is not due to RNA stability, as the level of the *nc1669-shf1* RNA was not reduced by the mutation (ii, Supplementary Figure S9B). Therefore, we concluded that the CSM is required for the full function of nc1669 to inhibit untimely mating during vegetative growth, through repression of premature expression of *ste11*.

## DISCUSSION

### ncRNA screening based on sequence, structure and biological tests

This study presents screening methods employing informatics focusing on conservation of the RNA structure, which was confirmed to be effective to identify functional ncRNAs through the biological screening. We screened for structurally conserved RNAs from 1857 annotated (including uncharacterized) ncRNAs, to find 151 structurally conserved ncRNAs, 14 of which were chosen for investigating their functions *in vivo*. As a result, we succeeded in identifying an ncRNA nc1669 functional in repression of untimely sexual differentiation. On the other hand, meiRNA, a well-characterized ncRNA required for progression of meiosis, was not detected in the screen. This is probably due to the screening strategy: ncRNAs well conserved in sequence and in secondary structure among relatives are selected *in silico*, and ncRNAs that are not highly conserved such as meiRNA may not be hit in the screen.

Prior to searching for conserved secondary structures, selection based on sequence conservation of ncRNAs in *S. pombe* and related species was performed at the initial step (see Figure 1B). This skips a huge amount of computational calculation which would be necessary if all the ncRNAs were searched for structural conservation. We compacted the screen to avoid the complexity in the computational operations by extracting only the conserved regions of *S. pombe* ncRNAs. This sequence-based screen in practice discarded 804 out of 1857 ncRNAs (see Figure 1B), although some ncRNAs that are conserved in structure but not in sequence could have been dismissed.

The detection sensitivity of structurally conserved ncRNAs estimated from well-known ncRNAs was ∼25% (see Figure 1C, D). There are two possible explanations for the reason why ∼75% were not detected as structurally conserved ncRNAs. First, even though a group of RNAs share functions over species, their structures are not necessarily unique in exerting these functions. The other possibility is that the RNAz software recognized that their structures are not sufficiently conserved. RNAz comprehensively assesses sequence similarity, structural similarity, covariance base substitution and thermodynamic stability to evaluate structural conservation (47). For example, RNAs with low thermodynamic stability are not selected even if their overall structures are similar over species, even though those might be structurally stabilized possibly through chemical modifications in cells, as seen in tRNAs (52). Such ncRNAs might be dismissed in our screen.

For ncRNA screening, the FDR was estimated to be ∼6% (Figure 1B). This was calculated through random shuffling of the fragments extracted by structure-based multiple alignment. Although FDR values calculated in this way may be underestimates, this accuracy is sufficient for our screening because, in any case, candidates next proceed to biological screening to test their phenotypes. Besides that, the FDR could be suppressed by increasing the selection threshold of RNAz (the cut-off of the RNA class probability *P*) to ≥0.9, which would exclude many candidates. When the cut-off of *P* was raised to ≥0.9 in our analysis, the FDR indeed decreased to 1.3%, but the detection sensitivity also decreased to 16% (Table 1), demonstrating that there is a trade-off between the FDR and detection sensitivity.

**Table 1.** FDR and detection sensitivity at different selection thresholds

	*P* ≥0.5	*P* ≥0.9
FDR	6.1%	1.3%
Detection sensitivity	25.7%	16.0%

Although many types of CSMs were found in our screen, it appears hard to find a consensus tendency in their structures (Figure 1E). Further computational analyses would be effective to find the structural tendency. For example, it is possible to classify CSMs based on their structural similarity (clustering analysis). For instance, software such as GraphClust (53), DotAligner (24) and RNAscClust (54) could be applicable for structure-based clustering of RNAs.

### nc1669 regulates initiation of sexual differentiation

In general, unconventional ncRNAs (excluding tRNA, rRNA, snRNA, snoRNA and miRNA) are classified into *cis*-acting and *trans*-acting, in which they function at proximal or distal gene loci, respectively. The majority of unconventional ncRNAs have been reported to function as *cis*-acting ncRNAs; in particular, they tend to regulate expression of nearby genes or overlapping antisense genes. nc1669 could regulate expression of *SPBTRNAGLU.08* and *SPNCRNA.1670* on the overlapping antisense strand, which respectively encode a tRNA and an ncRNA (Figure 3D). Phenotypic analyses may provide a hint to distinguish the way in which nc1669 acts.

The hyper-mating phenotype of *nc1669FL*Δ cells indicated that the ncRNA negatively regulates untimely initiation of sexual differentiation in nutrition-rich conditions (Figure 3A–C). In fission yeast, initiation of sexual differentiation during vegetative growth is negatively regulated by Pat1 kinase (55–57). The homothallic (*h*^90^) *pat1-114* temperature-sensitive mutant in rich media undergoes haploid meiosis at high temperature (34°C), whilst the mutant undergoes hyper-mating (but not haploid meiosis) at the semi-restrictive temperature of 29.5°C (55,56).

The cell fate of the *pat1-114* mutant is known to be determined by Ste7 protein. The *ste7*Δ mutant cannot initiate mating under nitrogen starvation, meaning that Ste7 promotes mating. At 29.5°C, *pat1-114 ste7*Δ cells initiate haploid meiosis, in contrast to hyper-mating of *pat1-114* cells (58). This suggests that Ste7 protein navigates *pat1-114* cells towards hyper-mating by blocking initiation of haploid meiosis at the semi-restrictive temperature. Although the *nc1669*Δ mutant exhibited hyper-mating, it never initiated haploid meiosis unlike *pat1-114*. It is therefore possible that nc1669 is involved in cell fate determination together with Ste7 to modulate the function of Pat1.

Two other major factors are known to inhibit untimely initiation of sexual differentiation in the presence of nutrients: TORC1 (target of rapamycin complex 1) and PKA (cAMP-dependent protein kinase) (59). TORC1 is a protein complex that contains the conserved TOR kinase Tor2. TORC1 is essential for cell growth and repression of sexual differentiation, as the *tor2* mutant undergoes mating in rich media (60–64). On the other hand, the cellular cAMP level varies depending on environmental conditions, and thus modulates the activity of its target, PKA (65,66). TORC1 and PKA signal transduction pathways synergistically repress the level of Ste11, the key transcription factor for sexual differentiation (49,59). In line with this, expression of *ste11* in vegetative conditions was up-regulated in the *nc1669*Δ mutant (i, Figure 4A). It is therefore possible that nc1669 somehow represses expression of *ste11* in rich media through the CSM, to block untimely initiation of sexual differentiation. The genetic loci of *nc1669* and *ste11* are close to each other on chromosome II (4109819–4107562 and 3983404–3987400, respectively), therefore it is possible that the nc1669 ncRNA may act in *cis* for repression of *ste11* transcription. This might account for the fact that expression of *nc1669* from plasmids did not fully complement the null mutant *nc1669FL*Δ (Figure 5A–C). It is alternatively possible that nc1669 may function together with the *rse1* ncRNA, which has been identified as a *cis*-acting long ncRNA that represses *ste11* expression (33).

It is also suggested that tRNAs or their related factors regulate sexual differentiation in budding and fission yeast (50,51). It was therefore possible that nc1669 acts *in cis*, to promote expression or subsequent modification of the encompassing tRNA gene *SPBTRNAGLU.08*. The *tRNAGlu08* gene on its own, however, appeared to be unrelated directly to sexual differentiation, because artificial expression of *tRNAGlu08* in *nc1669*Δ cells failed to suppress the hyper-mating phenotype (Figure 6D).

The target genes of nc1669 could be limited to factors related to sexual differentiation, as the *nc1669* knockout mutant did not display any other phenotypes in the biological screen. It remains elusive why each colony of *nc1669* mutants displayed a variable degree of the hyper-mating phenotype (Figure 3A, B). As we excluded possibilities of additional mutations through backcrossing, we interpret this as follows: the degree of the hyper-mating phenotype might be variable when the *nc1669* gene was first deleted in each cell. Since then, the degree of the phenotype in each cell could be maintained over generations inside single colonies in an epigenetic manner.

### Perspective

We have constructed gene deletion mutants for each of 12 ncRNAs that do not overlap with protein-coding regions, and systematically examined their phenotype. Although only the mutant of *SPNCRNA.1669* exhibited a remarkable phenotype, some other ncRNAs would be worth noticing. For instance, knockout of *SPNCRNA.491* resulted in moderate sensitivity to the microtubule poison TBZ. Besides that, RNA-seq profiling indicated that *SPNCRNA.510* is highly expressed (120 copies/cell) during vegetative growth, in comparison with the average transcript level (∼0.2–1 copy/cell) (67). Raman spectroscopy in combination with RNA-seq identified *SPNCRNA.510* as a gene whose expression level varied widely depending on the cultivation conditions (68).

Systematic deletions of 141 *S. pombe* long intergenic ncRNAs have been conducted to present functional profiling of those ncRNAs under ∼140 environmental conditions (37). ncRNA475, 507, 515, 808 and 965 in our list overlapped with theirs regarding phenotypes in stress conditions and in cell cycle progression. This may suggest that ncRNAs conserved in sequence and in structure are often functional under some environmental conditions.

We propose that such existing data for functional profiling and expression levels in various conditions (37,69), as well as in the mutants of RNA metabolic pathways (70), can be combined with our *in silico*/*in vivo* screening, so that functional ncRNAs that react to environmental changes can be efficiently highlighted. It would also be informative as a next step to determine the *in vitro* or *in vivo* secondary structure of the elicited ncRNAs using methods such as DMS-MaPseq (71).

During the plate-based *in vivo* screen, only a few genes displayed phenotypes in the conditions we tested. Parameters and thresholds in the *in silico* procedures may be further modulated to broaden the scope of candidates for the next *in vivo* step. It is also possible to flexibly design experimental conditions of the *in vivo* screening, based on the cellular events of interest, or on the expression patterns of individual candidates. These combinatory multi-omics studies might efficiently pinpoint their phenotypes to reveal elusive functions of uncharacterized ncRNAs.

## DATA AVAILABILITY

The datasets generated in this study can be downloaded as a supplementary datafile.

## Supplementary Material

gkac825_Supplemental_Files
