# Supplementary material for: Structure-based screening for functional non-coding RNAs in fission yeast identifies a factor repressing untimely initiation of sexual differentiation
Source: Nucleic Acids Res. 2022 Oct 19;50(19):11229–42. doi: 10.1093/nar/gkac825 (PMC9638895; doi:10.1093/nar/gkac825)
Supplement: gkac825_Supplemental_Files [file gkac825_supplemental_files.zip › (REV)_Supplementary information file_revision220907F.pdf]

Supplementary information

Structure-based screening for functional non-coding RNAs in fission yeast identifies a factor repressing untimely initiation of sexual differentiation

Yu Ono, Kenta Katayama, Tomoki Onuma, Kento Kubo, Hayato Tsuyuzaki, Michiaki Hamada, Masamitsu Sato

Supplementary tables S1, S2
Supplementary figures S1–S9

Supplementary table S1. *S. pombe* strains used in this study

Strain Genotype Figures

Strain	Genotype	Figures
JY3	h^{90} (wild-type)	2D 3A 3B 3C 4A 5B 5C 6B 6C 6D 6F 6G 6H S1 S2 S3 S4 S5 S6 S7 S8 S9
MJ0006	h^{90} *alp7::ura4*$^{+}$ *leu1 ura4 ade6-M216*	2D S2 S3 S4
MJ1551	h^{90} *pap1::ura4*$^{+}$ *leu1 ura4 ade6-M216*	S5 S6 S7
SY50	h^{90} *rad3::ura4*$^{+}$ *ura4-D18*	2D S2 S3 S4
KEN0137	h^{90} *sty1::kanR*	S5 S6 S7
KEN0134	h^{90} *SPNCRNA.192::kanR*	2D S1 S2 S3 S4 S5 S6 S7 S8
KEN0135	h^{90} *SPNCRNA.192::kanR*	S8
KEN0128	h^{90} *SPNCRNA.254::kanR*	2D S1 S2 S3 S4 S5 S6 S7 S8
KEN0129	h^{90} *SPNCRNA.254::kanR*	S8
KEN0175	h^{90} *SPNCRNA.475::kanR*	S1 S2 S3 S4 S5 S6 S7 S8
KEN0176	h^{90} *SPNCRNA.475::kanR*	S1 S2 S3 S4 S5 S6 S7 S8
KEN0177	h^{90} *SPNCRNA.475::kanR*	S8
KEN0146	h^{90} *SPNCRNA.491::kanR*	S1 S2 S3 S4 S5 S6 S7 S8
KEN0147	h^{90} *SPNCRNA.491::kanR*	S8
KEN0148	h^{90} *SPNCRNA.507::kanR*	S1 S2 S3 S4 S5 S6 S7 S8
KEN0149	h^{90} *SPNCRNA.507::kanR*	S8
KEN0178	h^{90} *SPNCRNA.510::kanR*	S1 S2 S3 S4 S5 S6 S7 S8
KEN0179	h^{90} *SPNCRNA.510::kanR*	S1 S2 S3 S4 S5 S6 S7 S8
KEN0180	h^{90} *SPNCRNA.510::kanR*	S8
KEN0154	h^{90} *SPNCRNA.515::kanR*	S1 S2 S3 S4 S5 S6 S7 S8
KEN0155	h^{90} *SPNCRNA.515::kanR*	S8
KEN0169	h^{90} *SPNCRNA.965::kanR*	S1 S2 S3 S4 S5 S6 S7 S8
KEN0170	h^{90} *SPNCRNA.965::kanR*	S1 S2 S3 S4 S5 S6 S7 S8
KEN0136	h^{90} *SPNCRNA.1094::kanR*	S1 S2 S3 S4 S5 S6 S7 S8
KEN0143	h^{90} *SPNCRNA.1094::kanR*	S8
KEN0172	h^{90} *SPNCRNA.1490::kanR*	S1 S2 S3 S4 S5 S6 S7 S8
KEN0173	h^{90} *SPNCRNA.1490::kanR*	S1 S2 S3 S4 S5 S6 S7 S8
KEN0152	h^{90} *SPNCRNA.1511::kanR*	S1 S2 S3 S4 S5 S6 S7 S8
KEN0153	h^{90} *SPNCRNA.1511::kanR*	S8
KEN0040	h^{90} *SPNCRNA.1669::kanR* #1	3A 3B 6H S1 S2 S3 S4 S5 S6 S7 S8
KEN0041	h^{90} *SPNCRNA.1669::kanR* #2	3A 3B 3C 4A 6B 6H S8
KEN0187	h^{90} *SPNCRNA.1669FL::kanR* #1	3B 6H
KEN0188	h^{90} *SPNCRNA.1669FL::kanR* #2	3B 4A 6B 6H
TO0005	h^{90} (wild-type) +pREP1*natR*	5B 5C 6C 6D 6F 6G
TO0006	h^{90} *SPNCRNA.1669FL::kanR* #2 +pREP1*natR*	5B 5C 6C 6D 6F 6G
TO0007	h^{90} *SPNCRNA.1669FL::kanR* #2 +pREP1*natR-SPNCRNA.1669FL*	5B 5C
TO0008	h^{90} *SPNCRNA.1669FL::kanR* #2 +pREP1*natR-SPNCRNA.1669* without CSM	6F 6G
TO0021	h^{90} *SPNCRNA.1669FL::kanR* #2 +pREP1*natR-SPBTRNAGLU.08*	6C 6D
TO0023	h^{90} *SPNCRNA.1669FL::kanR* #2 +pREP1*natR-SPNCRNA.1669* without *SPNCRNA.1670*	5B 5C
YO0619	h^{90} *SPNCRNA.1669-shf1*	6H S9

SY50 was provided by the National Bioresource Project (NBRP), Japan. JY3, MJ0006 and MJ1551 are our laboratory stock. Other strains were made in this study.

Supplementary table S2. Oligonucleotides used in this study

<u>For construction of gene deletion mutants (5′-3′)</u>

for KEN0137

P1 AAAGCTGGCTTAACAACTTTACC
P2 TTAATTAACCCGGGGATCCGTTTATTCAAACTGGTTACAAAAAGGAC
P3 GTTTAAACGAGCTCGAATTCACAAGTTGTAGATAAAGCCTTAAAAGTTG
P4 ATAAAACCCGTAACTACATTAACGAAC

for KEN0134, KEN0135

P1 ACAGTATGTTAATGAGTATGAGAGTTGG
P2 TTAATTAACCCGGGGATCCGGGAATGATCTATTTTGAAATGAAAGCG
P3 GTTTAAACGAGCTCGAATTCGGTGTATCAATTACTTCTGCAATCC
P4 GATAGCCATAAGGAATATTCTGGTATCC

for KEN0128, KEN0129

P1 TAAAGAGGATTCCTTGTAAATGAATCG
P2 TTAATTAACCCGGGGATCCGCCATTACAATGCCTTTATACAACTATCCG
P3 GTTTAAACGAGCTCGAATTCGCAATAAATCAGTGGAATATCGTGTTGG
P4 GAGACATAAACGAAATAACGCTGAACG

for KEN0175, KEN0176, KEN0177

P1 GTGCAAAAAGGATGTATGAACTTAGAGG
P2 TTAATTAACCCGGGGATCCGCTCAGAGAAGTAGAACTTTGTTGTAGCC
P3 GTTTAAACGAGCTCGAATTCGGGTGCAACTTTCAAATTATTCTAAAGGG
P4 AAGAAATAGTCATTCTCCCAATCCTCG

for KEN0146, KEN0147

P1 TTGTGTTCCTCCTCATTCATACGCC
P2 TTAATTAACCCGGGGATCCGCGTAATTTAACGATATAAGCCGTATTAGG
P3 GTTTAAACGAGCTCGAATTCAATGACGATAATGGATCCAGATGG
P4 CAAATCTGCAGCAATCAACGTTGCC

for KEN0148, KEN0149

P1 GGTGAAATGGAGCGTGATTGTTTGATTGC
P2 TTAATTAACCCGGGGATCCGGTTGTGTAAAGAATAATAAAGCATATGGC
P3 GTTTAAACGAGCTCGAATTCCGAATGTTGTTCTGAGATTTTGTTTCG
P4 GCGTCAGCATAACTTCTTTCTTCAAGCC

for KEN0178, KEN0179, KEN0180

P1 CAACAATGCTTTAATGGTGGAGGTTGG
P2 TTAATTAACCCGGGGATCCGATGTGGAAGGATTAGTTTTCGCACACC
P3 GTTTAAACGAGCTCGAATTCCCTGCACACCTTGTTACGTTTATTGGC
P4 CTTGAAATTTCTTTCTACCTTTTTGCTTCC

for KEN0154, KEN0155

P1 TCGTCTTGCTAAGTAAGTCTTGCGTGG
P2 TTAATTAACCCGGGGATCCGGGGATTCTTGTAGATGAATAGTAAGC
P3 GTTTAAACGAGCTCGAATTCCGTGGAGACAGTCTTGATAGTAGTGTGC
P4 CTGTATGTTAGGAATACGAACGTAGGC

for KEN0169, KEN0170

P1 TGTTGCTCATATATACGCACAGAATGG

P2 TTAATTAACCCGGGGATCCGAGCACACAAAGCAAGTAAACTAGTTGG
P3 GTTTAAACGAGCTCGAATTCTATACTTGAACGATCGGCTATTTACAGG
P4 ACACTCTTCATCGGCTTCACTTCCTCG

for KEN0136, KEN0143
P1 GTCCAAGGAACTATGTAAAAGGAGTGG
P2 TTAATTAACCCGGGGATCCGGCAACTCGCTTTCCAACTTATCCTTGC
P3 GTTTAAACGAGCTCGAATTCAGTGGTAACAACAGATCAACAGCCG
P4 GGATCAACCAGGTAACTATCGTTAGAGG

for KEN0172, KEN0173
P1 CTCTTCTTCGCTTTCAATCTCATCTTCG
P2 TTAATTAACCCGGGGATCCGGCGATGACTGTAAAGAATAAAACTGGCG
P3 GTTTAAACGAGCTCGAATTCGCTTAGATTTATGTAAGTTGAATGTGGG
P4 CACTCCATTACCAATCCTTTATCAATACG

for KEN0152, KEN0153
P1 TATGAACATCGTGCCAATTGCAGTGCC
P2 TTAATTAACCCGGGGATCCGAGGGTAGGGTTGTGACTACCTTAACG
P3 GTTTAAACGAGCTCGAATTCCTGAAAATAGTAAAGTGCCAGTGTGG
P4 TAAGTTGCTTTCAAGACTTCCTGTTGC

for KEN0040, KEN0041
P1 TAGTTATCAGGCAAGAGAGAACTGG
P2 TTAATTAACCCGGGGATCCGCATATCACCTAGTTTCGTCCATACC
P3 GTTTAAACGAGCTCGAATTCAAAGAGGACAAGACAGGACAAGG
P4 GTCAATTGGATTTTTTGGTATTTTGGG

for KEN0187, KEN0188
P1 same as KEN0040, KEN0041
P2 same as KEN0040, KEN0041
P3 GTTTAAACGAGCTCGAATTCACCTGCTATGTAAATAGCCTAACAGACG
P4 GTAAAGAGTATATGGGGACAGCGTATCC

For construction of plasmids

for pREP1*natR*
GGCGAGTCTAACTCCTTAACCAAGACAGAATAAGTCATCAGCG
GTGAATGCTGGTCGCTATACTGGCTTGGCGTAATCATGGTCATAGC
CGCTGATGACTTATTCTGTCTTGGTTAAGGAGTTAGACTCGCC
GCTATGACCATGATTACGCCAAGCCAGTATAGCGACCAGCATTCAC

for pREP1*natR-SPNCRNA.1669FL*
CCAAGAAGTAGTCTCAGAGCATGTATCAATGGGATGCCAATGG
CCATTGGCATCCCATTGATACATGCTCTGAGACTACTTCTTGG
GGCTACTGGATGGTTCAGTCACAGGCGATAGTGGGAGAAGC
GCTTCTCCCACTATCGCCTGTGACTGAACCATCCAGTAGCC

for pREP1*natR-SPNCRNA.1669* without *SPNCRNA.1670*
CTACATAACCACTCTCAAACTCACCTTTAACAGCCGAAAACTTAAGACG
CGTCTTAAGTTTTCGGCTGTTAAAGGTGAGTTTGAGAGTGGTTATGTAG

for pREP1*natR-SPBTRNAGLU.08*
CGTCTAAGGATACAGAAGATTGTTCGGCAAAGTCGCATGTTAG
CCAAGAAGTAGTCTCAGAGCAGATGCCAATGGATGCTTAGC

GCTAAGCATCCATTGGCATCTGCTCTGAGACTACTTCTTGG
CTAACATGCGACTTTGCCGAACAATCTTCTGTATCCTTAGACG

for pREP1*natR-SPNCRNA.1669* without CSM
GCCCAAAATACCAAAAAATCCAATGTCGACGAAATAACCCCAATGACC
GGTCATTGGGGTTATTTCGTCGACATTGGATTTTTTGGTATTTTGGGC

For Colony PCR

	Strain	Sequence (5′-3′)
Pch1	common	CGACATCATCTGCCCAGATGCG
Pch2	KEN0137	ATTTAACTTCATGTACACAAGATGTCC
	KEN0134, KEN0135	AACAGTTAGCAGAGGATCACCACACG
	KEN0128, KEN0129	GAAGATTTGAACAGGAAGGAGACTGGG
	KEN0175, KEN0176, KEN0177	ACTGATATTTTAGCAACACACTATGCG
	KEN0146, KEN0147	ACAATGAATATCACTCTCTTTTAATGG
	KEN0148, KEN0149	GATAGTATTCAGTTTTGCAAACCTGCC
	KEN0178, KEN0179, KEN0180	GAAGCCAGGAACTTAATCTTTACTAACC
	KEN0154, KEN0155	ATTGAAAGTATGCTTCGAATTGACGC
	KEN0169, KEN0170	TGAAAGAATACTGGAGATTTGATTCACG
	KEN0136, KEN0143	CGATAACTGATTTAATGAGCCATTCGC
	KEN0172, KEN0173	ATATACAAGGAGACTGTGTTTCACCTCC
	KEN0152, KEN0153	ATTCGTTATGGTGGTGCTAAGTCGTGG
	KEN0040, KEN0041	CATAGCAGGTGATCAGGTCATTGG
	KEN0187, KEN0188	AAAGATGTGACGGCAATGAATGGG

For strand-specific reverse transcription (5′-3′)
act1: GTCACGAACAATTTCACGTTCGG
tRNAGlu08: CCGTCAGGGGGAATCGAAC
nc1669: CGGTTGATGGTTGCTCAGAAATAAC
nc1670: CGTTCCTTACATTCTACAACGTC
ste11: GAAGGTCCCAATGAGTTAGCATC

For quantitative real-time PCR (5′-3′)
for *nc1669* (**Figure 5C**, **6F**)
TCGTTCCATGCTAACGATTCGC
TTCAGCGCCTCTTTCTATTCCG

for *nc1669* (**Figure 4A, S9B**)
GGGAAGGAGGACAAATTTGTAAATAG
GCAGATACCCGATTGTGTCC

for *nc1670*
CGTCGTACCCTCAACATAGTAC
GGTATGGGTCAATTAGCATGCTAC

for *tRNAGlu08*
CCAGTGGCTAGGATTCATCGC
CGTCAGGGGGAATCGAACC

for *ste11*
CGTATTTATACGGCCAAGAGACCG
GACGAAGAGACGCATGTATAGCC

for *act1*

TGAGGAGCACCCTTGCTTGT
TCTTCTCACGGTTGGATTTGG

The CSM sequence of the WT nc1669 RNA (5′-3′)
UGACUACAUACAUAUUUUUGAUAAAAAUACUCCGUCAGGGGGAAUCGAACCCCCGCC
GCAUCGGUGAGAGCGAUGAAUCCUAGCCACUGGACCAUGACGGAUUUAGUUAUAUA
CCGA

The CSM-*shf1* sequence (5′-3′, the underline indicates the *Nde*I restriction site)
ACUUAACAUAAUCAUGCGCGCACAGAAAUGGGUAAGUGGGGACGGGUCAGUAUUUC
CGGCCGCUAGACACUUUUUAAGCCUACAACAUCCUGAACGCAUAUGGGCCCACAUUA
UUAA

The sgRNA target sequence (crRNA) in DNA (5′-3′)

TTTGATAAAAATACTCCGTC

The primer pair for colony PCR to amplify the region including CSM-*shf1* (5′-3′)

CCTACAACATCCTGAACGCATATG (specific to the CSM-*shf1* mutant sequence)

GTAAAGAGTATATGGGGACAGCGTATCC (originated from the *nc1669* WT sequence)

Supplementary Figures

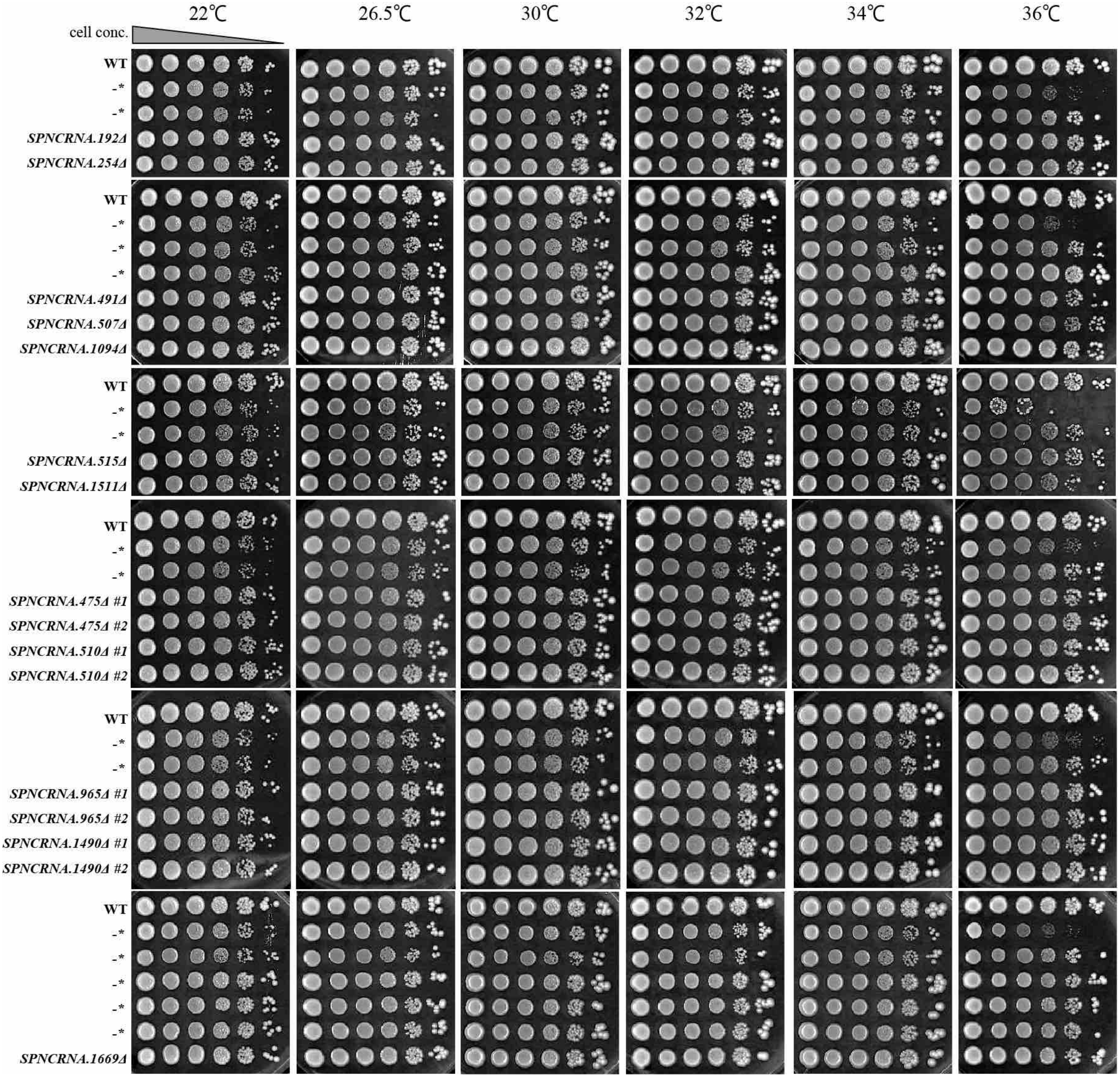

Figure S1. Cell viability assay for ncRNA deletion mutants under various temperatures

Cells of ncRNA deletion mutants were sequentially diluted and spotted on YE5S plate. Then, cells were incubated at 22˚C, 26.5˚C, 30˚C, 32˚C, 34˚C and 36˚C. 26.5˚C and 30˚C are the standard temperatures for growing *S. pombe* cells. #1 and #2 indicates distinct clones of the same genotype. * Strains with asterisks are not related to this experiment.

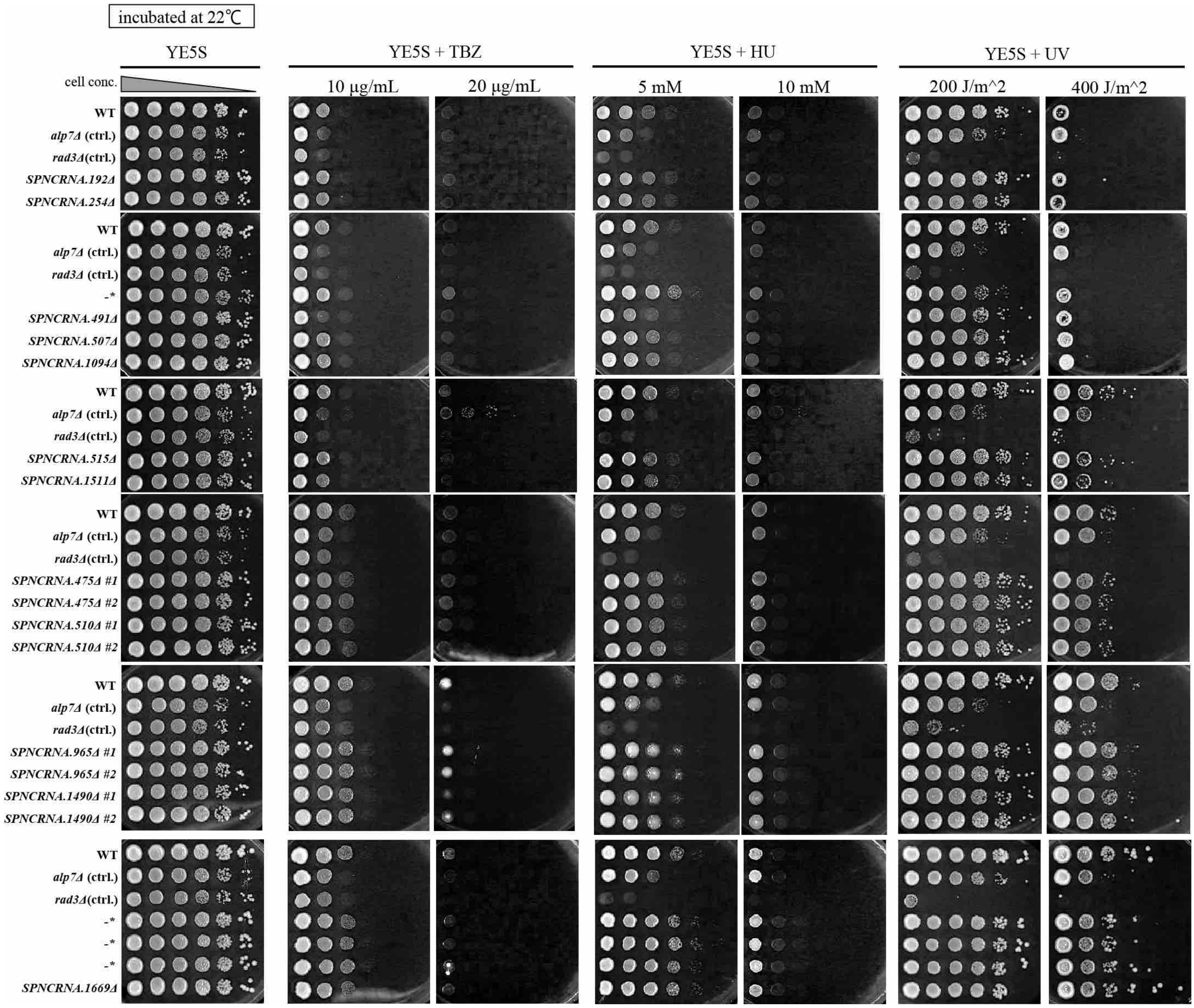

Figure S2. Cell viability assay for ncRNA deletion mutants under TBZ, HU and UV at 22˚C

Cells of ncRNA deletion mutants were sequentially diluted and spotted on YE5S, YE5S+TBZ (thiabendazole) or YE5S+HU (hydroxyurea) plate. In addition, YE5S plate which was irradiated with UV after spotting is denoted as YE5S+UV. Then, cells were incubated at 22˚C. *alp7*Δ mutant was used as TBZ sensitivity control. *rad3*Δ mutants was used as HU and UV sensitivity control. #1 and #2 indicates distinct clones of the same genotype. Regarding Rad3, it was previously reported that *rad3*Δ cells showed sensitivity to microtubule poisons including TBZ (1). This could be due to a delay of entry into mitosis through defective cytoskeleton, which activated Rad3-dependent checkpoint. * Strains with asterisks are not related to this experiment.

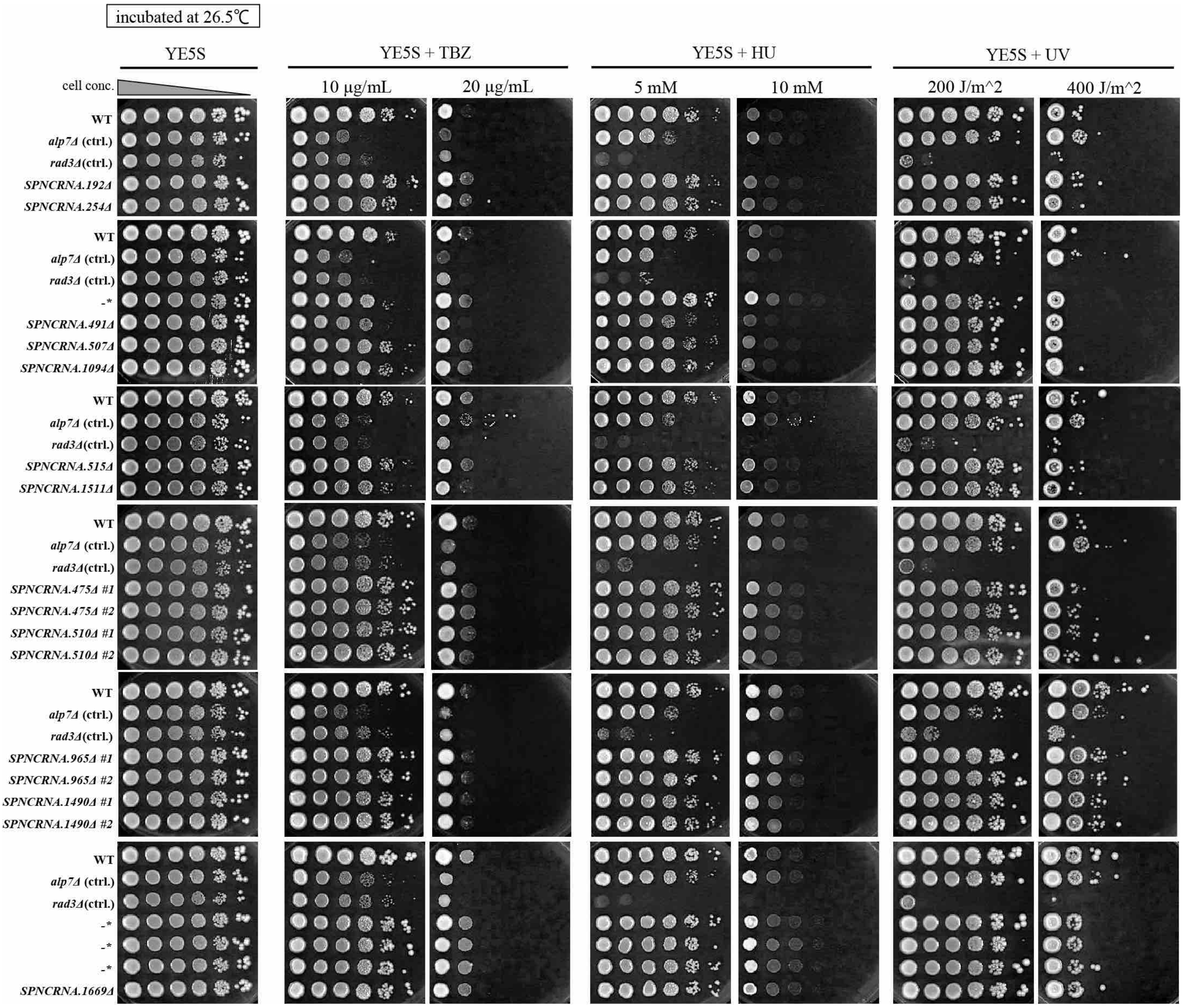

Figure S3. Cell viability assay for ncRNA deletion mutants under TBZ, HU and UV at 26.5˚C

Cells of ncRNA deletion mutants were sequentially diluted and spotted on YE5S, YE5S+TBZ (thiabendazole) or YE5S+HU (hydroxyurea) plate. In addition, YE5S plate which was irradiated with UV after spotting is denoted as YE5S+UV. Then, cells were incubated at 26.5˚C. *alp7*Δ mutant was used as TBZ sensitivity control. *rad3*Δ mutants was used as HU and UV sensitivity control. #1 and #2 indicates distinct clones of the same genotype. Some panels on the top are also shown in **Figure 2D** as representatives. * Strains with asterisks are not related to this experiment.

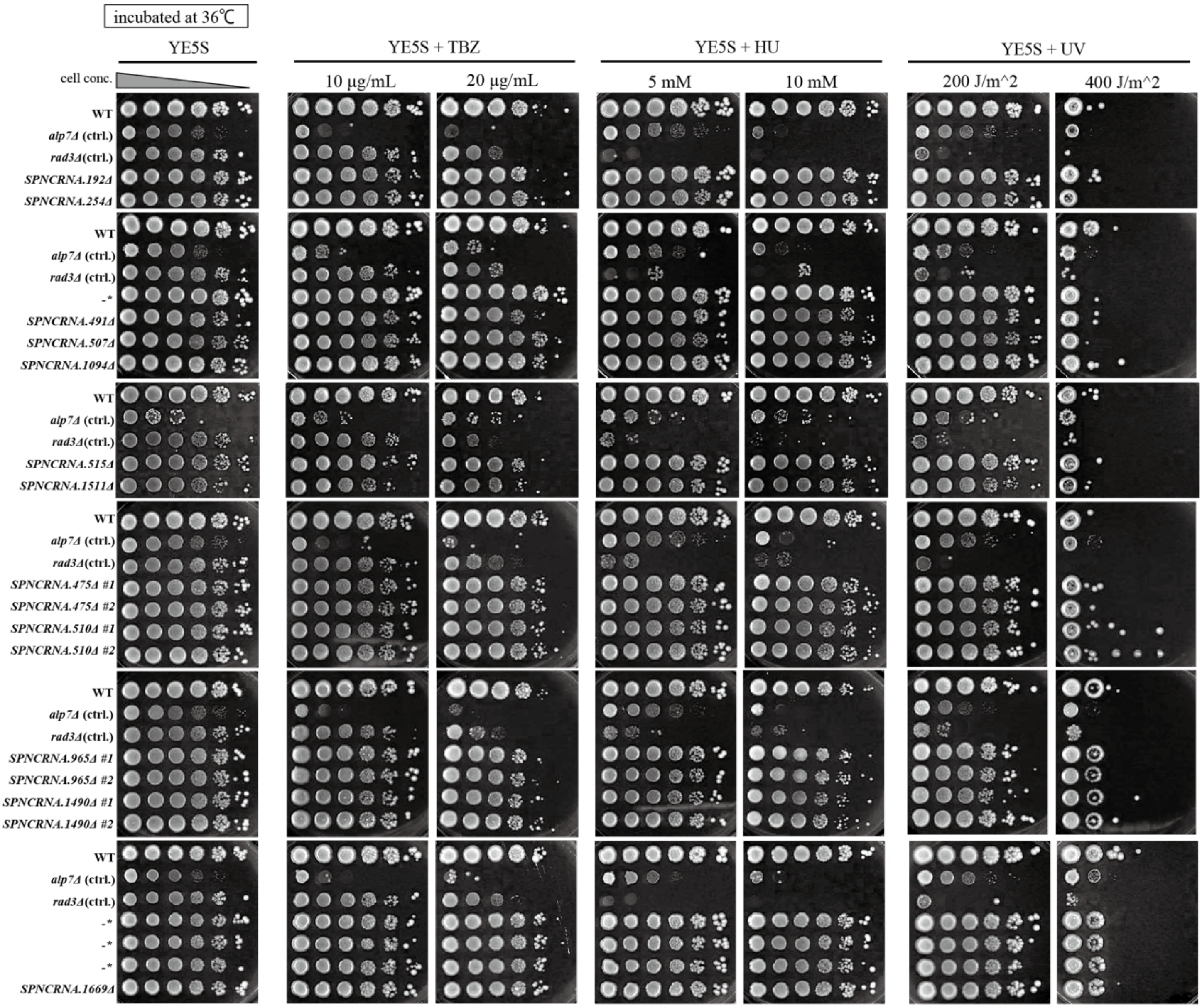

Figure S4. Cell viability assay for ncRNA deletion mutants under TBZ, HU and UV at 36˚C

Cells of ncRNA deletion mutants were sequentially diluted and spotted on YE5S, YE5S+TBZ (thiabendazole) or YE5S+HU (hydroxyurea) plate. In addition, YE5S plate which was irradiated with UV after spotting is denoted as YE5S+UV. Then, cells were incubated at 36˚C. *alp7*Δ mutant was used as TBZ sensitivity control. *rad3*Δ mutants was used as HU and UV sensitivity control. #1 and #2 indicates distinct clones of the same genotype. * Strains with asterisks are not related to this experiment.

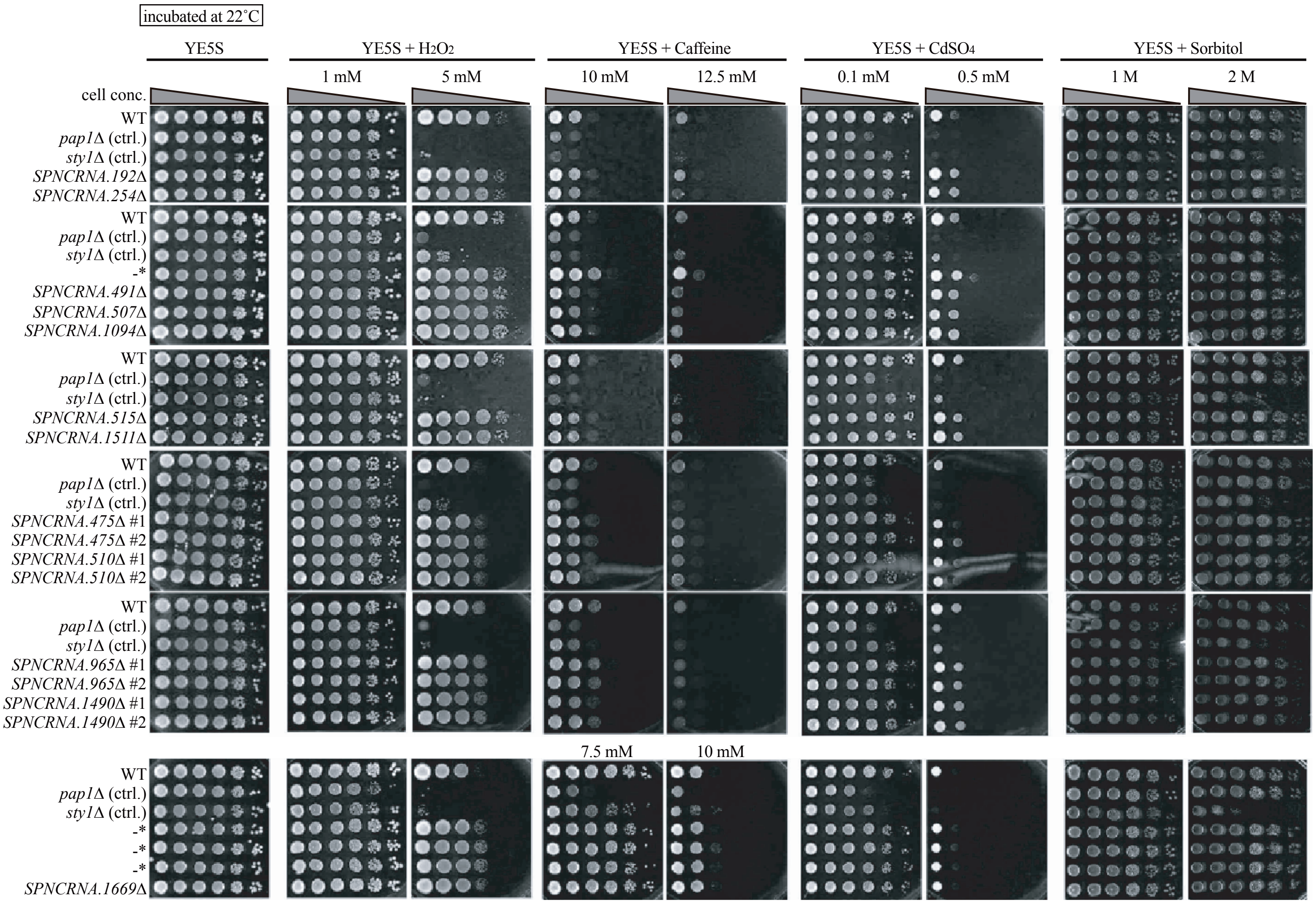

Figure S5. Cell viability assay for ncRNA deletion mutants under H_2O_2, caffeine, $CdSO_4$ and sorbitol at 22˚C

Cells of ncRNA deletion mutants were sequentially diluted and spotted on YE5S, YE5S+H_2O_2, YE5S+caffeine, YE5S+$CdSO_4$ or YE5S+sorbitol plate. Then, cells were incubated at 22˚C. *pap1*Δ and *sty1*Δ mutants were used as H_2O_2, caffeine and $CdSO_4$ sensitivity control. In addition, *sty1*Δ mutant was also used as sorbitol sensitivity control. #1 and #2 indicates distinct clones of the same genotype. * Strains with asterisks are not related to this experiment.

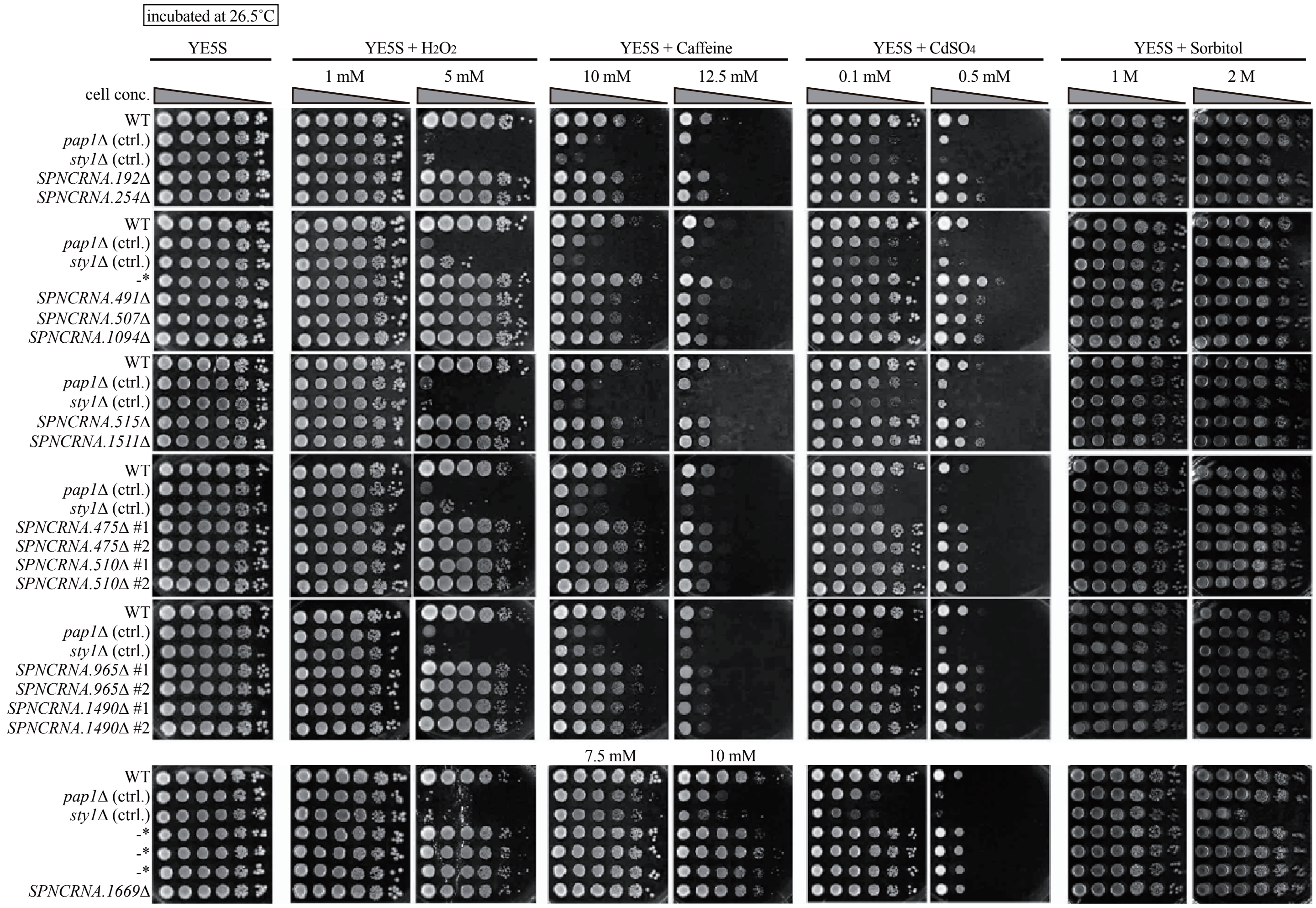

Figure S6. Cell viability assay for ncRNA deletion mutants under H_2O_2, caffeine, $CdSO_4$ and sorbitol at 26.5˚C

Cells of ncRNA deletion mutants were sequentially diluted and spotted on YE5S, YE5S+H_2O_2, YE5S+caffeine, YE5S+$CdSO_4$ or YE5S+sorbitol plate. Then, cells were incubated at 26.5˚C. *pap1*Δ and *sty1*Δ mutants were used as H_2O_2, caffeine and $CdSO_4$ sensitivity control. In addition, *sty1*Δ mutant was also used as sorbitol sensitivity control. #1 and #2 indicates distinct clones of the same genotype. * Strains with asterisks are not related to this experiment.

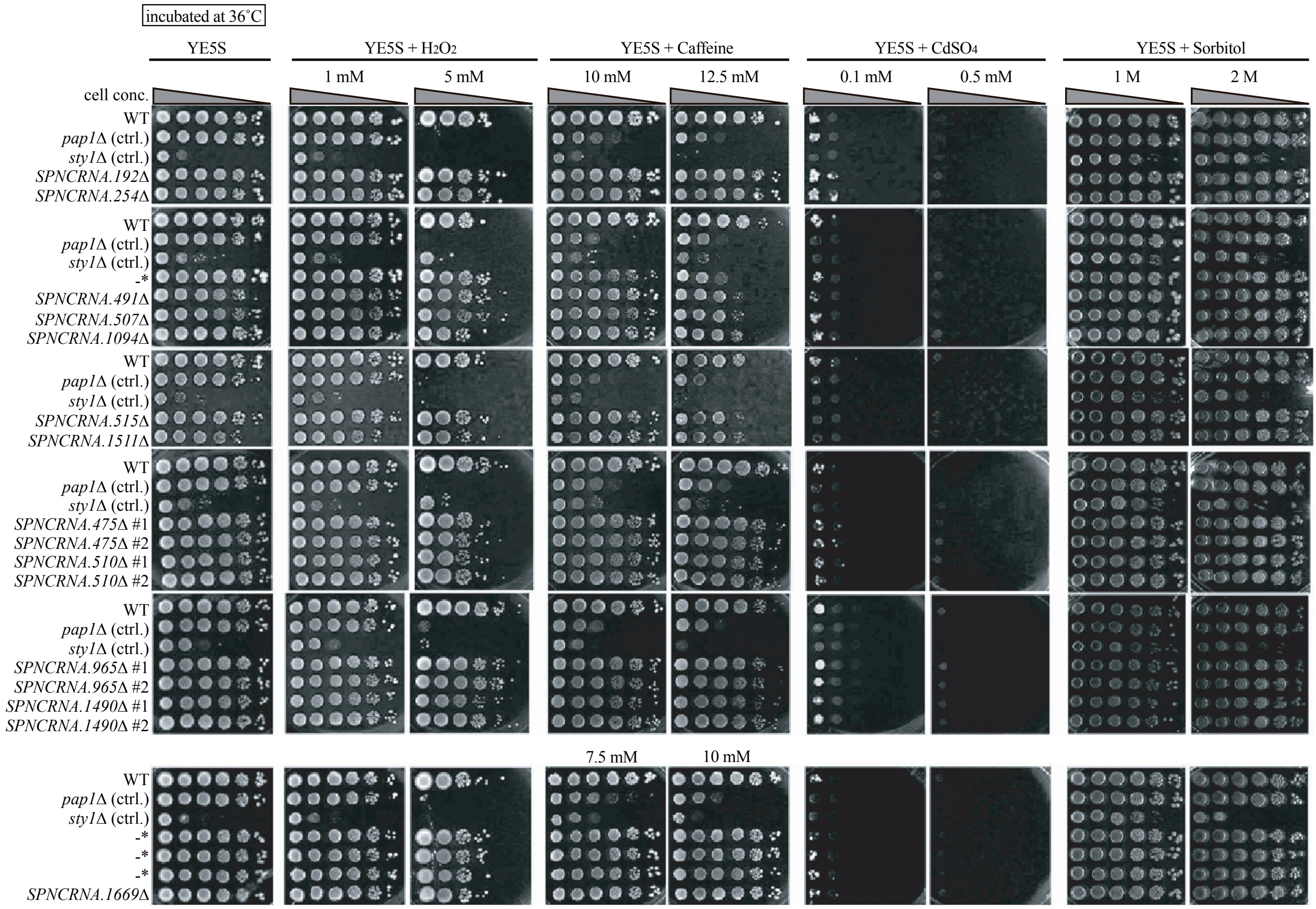

Figure S7. Cell viability assay for ncRNA deletion mutants under H_2O_2, caffeine, $CdSO_4$ and sorbitol at 36˚C

Cells of ncRNA deletion mutants were sequentially diluted and spotted on YE5S, YE5S+H_2O_2, YE5S+caffeine, YE5S+$CdSO_4$ or YE5S+sorbitol plate. Then, cells were incubated at 36˚C. *pap1*Δ and *sty1*Δ mutants were used as H_2O_2, caffeine and $CdSO_4$ sensitivity control. In addition, *sty1*Δ mutant was also used as sorbitol sensitivity control. #1 and #2 indicates distinct clones of the same genotype. * Strains with asterisks are not related to this experiment.

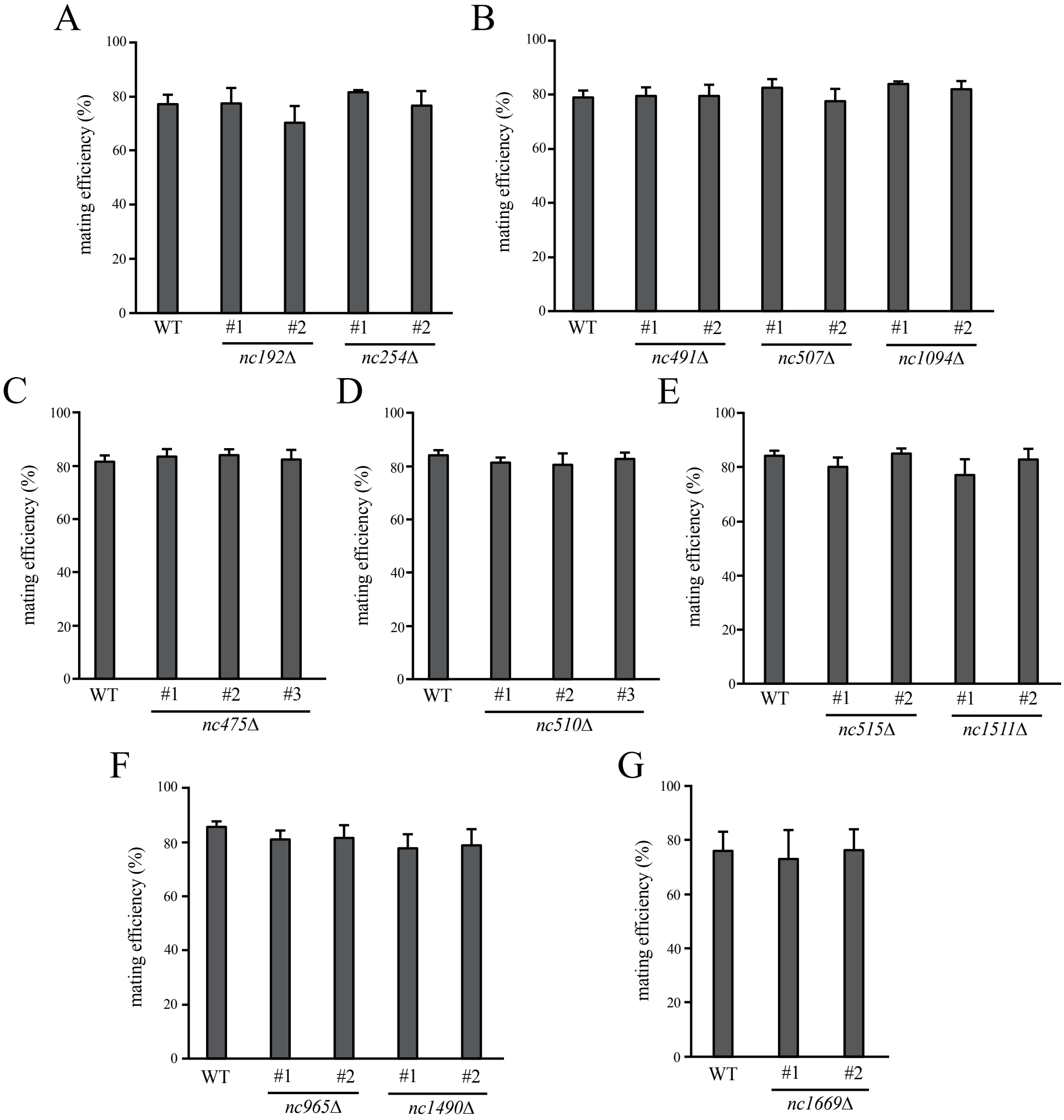

Figure S8. Mating efficiency of ncRNA deletion mutants at nitrogen starved condition

(**A–G**) Each ncRNA deletion mutant and the WT strain were grown on SPA plate at 26.5°C for 24 h. Multiple clones, denoted as #1, #2 and #3 of the same genotype, were subjected to experiments. Mating efficiency was observed. mating efficiency = 2 x number of zygotes / (2 x number of zygotes + number of unmated cells). Average ± SEM (3 trails).

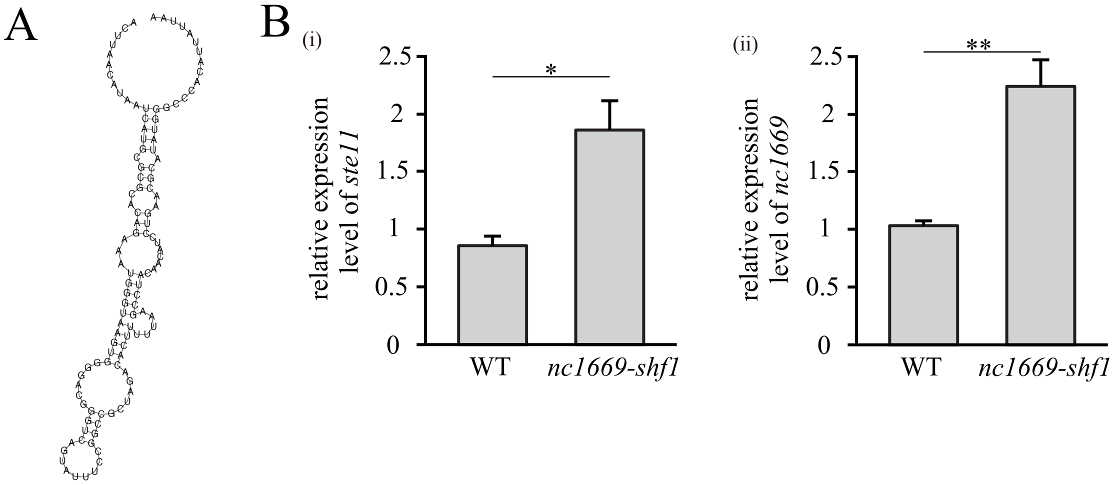

Figure S9. Secondary structure of CSM-*shf1* and expression levels of *ste11* and *nc1669* in the *nc1669-shf1* mutant

(**A**) Secondary structure of CSM-*shf1* predicted by RNAalifold (2). (**B**) The relative expression level of *ste11* (i) and the nc1669 ncRNA (ii) in WT and in *nc1669-shf1* mutant cells in EMM+N medium. Average ± SEM (3 trails). *p = 0.0113 (i), **p = 0.00784 (ii) (Student's t-test).

References for Supplementary Information

1. Herring,M., Davenport,N., Stephan,K., Campbell,S., White,R., Kark,J. and Wolkow,T.D. (2010) Fission yeast Rad26ATRIP delays spindle-pole-body separation following interphase microtubule damage. *J Cell Sci*, **123**, 1537–1545.

2. Bernhart,S.H., Hofacker,I.L., Will,S., Gruber,A.R. and Stadler,P.F. (2008) RNAalifold: improved consensus structure prediction for RNA alignments. *BMC Bioinformatics*, 9, 474–474.
